# Extracellular vesicles from colorectal cancer cells promote metastasis via the NOD1 signalling pathway

**DOI:** 10.1002/jev2.12264

**Published:** 2022-09-06

**Authors:** Xiduan Wei, Jingjia Ye, Yameng Pei, Chunting Wang, Hongzhen Yang, Jingyuan Tian, Guangxu Si, Yao Ma, Kun Wang, Gang Liu

**Affiliations:** ^1^ School of Pharmaceutical Sciences Tsinghua University Beijing P. R. China; ^2^ Institute of Materia Medica Chinese Academy of Medical Sciences & Peking Union Medical College Beijing P. R. China; ^3^ Key Laboratory of Carcinogenesis and Translational Research (Ministry of Education/Beijing), Department of Hepatobiliary and Pancreatic Surgery Unit I Peking University Cancer Hospital & Institute, Beijing; ^4^ Key laboratory of Molecular Pharmacology and Drug Evalution (Yantai University), Ministry of Education; Collaborative Innovation Center of Advanced Drug Delivery System and Biotech Drugs in Universities of Shandong Yantai University Yantai P. R. China

**Keywords:** colorectal cancer, extracellular vesicles, metastatic liver, NOD1 signalling

## Abstract

Pattern‐recognition receptors (PRRs) have been shown to promote tumour metastasis via sensing tumour cell‐derived small extracellular vesicles (EVs). Nucleotide‐binding oligomerisation domain 1 (NOD1), a cytoplasmic PRR, plays a role in colorectal cancer (CRC) by detecting bacterial products. However, the precise mechanisms underlying the effects of NOD1, following identification of CRC cell‐derived EVs (CRC‐EVs), to potentiate CRC liver metastasis (CRC‐LM), remain poorly understood. Here, we demonstrate that CRC‐EVs activate NOD1 in macrophages to initiate secretion of inflammatory cytokines and chemokines. NOD1‐activated macrophages also promote CRC cell migration, while in a murine model of liver metastasis (LM), NOD1‐deficient mice exhibit reduced metastasis following CRC‐EV treatment. Furthermore, cell division cycle 42 (CDC42), a small Rho guanosine‐5′‐triphosphate (GTP)ase, is delivered by CRC‐EVs into macrophages where it activates NOD1. In addition, EVs from the plasma of patients with CRC‐LM mediate NOD1 activation in human peripheral blood mononuclear cells. Moreover, high NOD1 expression in tumour tissues is associated with poor prognosis of CRC‐LM. Our findings suggest that CRC‐EVs activate NOD1 to promote tumour metastasis, thus, NOD1 may serve as a potential target in the diagnosis and treatment of CRC‐LM.

## 1 INTRODUCTION

Tumour metastasis is the largest contributor to cancer‐related mortality (Steeg, 2016). Hence, an urgent need exists for the prevention of tumour metastasis. This is particularly true for colorectal cancer (CRC), which is the second most deadly cancer worldwide, with approximately 940,000 deaths estimated for 2020 (Sung et al., 2021) and for which liver metastases (LM) are observed in nearly half of the patients during their disease course (Bird et al., Majeed, 2006; Slesser et al., 2013). CRC‐LM are associated with poor prognosis, with overall survival durations of 6–12 months if left untreated (Al Bandar & Kim, 2017). Thus, clarification of the mechanisms driving LM in CRC and identification of effective markers for CRC‐LM diagnosis and therapeutic targets for preventing metastasis, are critical for the effective control of CRC‐LM.

Chronic inflammation and infection are distinct factors that initiate the development of CRC and facilitate CRC‐LM progression (Keum & Giovannucci, 2019). Immune and inflammatory responses in infectious and inflammatory diseases are orchestrated by pattern‐recognition receptors (PRRs), which recognise pathogen‐associated molecular patterns (PAMPs) or danger‐associated molecular patterns (DAMPs) (Bianchi, 2007; Janeway & Medzhitov, 2002). Nucleotide‐binding oligomerisation domain (NOD)‐containing protein 1(NOD1), a host intracellular PRR, recognises bacterial peptidoglycan component diaminopimelic acid(DAP) to initiate an inflammatory response by interacting with receptor‐interacting protein 2 kinase (RIP2) to trigger pathways downstream of nuclear factor (NF)‐κB and mitogen‐activated protein kinase (MAPK) (Keestra‐Gounder & Tsolis, 2017). NOD1 activation reportedly fosters an immunosuppressive tumour microenvironment to promote CRC development in both colitis‐associated carcinogenesis and spontaneous carcinogenesis (Maisonneuve et al., 2021). Moreover, NOD1 activation by C12‐iE‐DAP (a highly selective NOD1 ligand) augments CRC cell adhesion and migration in vitro and in vivo, and promotes LM in a murine CRC‐LM model (Jiang et al., 2020). However, the mechanisms by which host NOD1 modulates CRC metastasis remain obscure.

Tumour cell‐derived extracellular vesicles (EVs), in which are smaller than 200 nm in diameter, consist mainly of exosomes, microvesicles or microparticles (Cocozza et al., 2020). EVs activate PRRs resulting in the induction of inflammatory responses; this contributes to premetastatic niche formation and promotes tumour cell metastasis (Tkach & Thery, 2016; Zhou et al., 2020). EVs contain proteins, lipids, RNA and DNA, working as a bridge in cell‐to‐cell communication (Kowal et al., 2014). PRRs, such as Toll‐like receptor (TLR) 2, TLR3 and TLR8, are activated by proteins (heat shock proteins 70 and 72), small nuclear RNA, and microRNAs (miRNA‐21 and miRNA‐29a) in tumour cell‐derived EVs, respectively; they mediate the function of the innate immune response to promote tumour metastasis (Chalmin et al., 2010; Fabbri et al., 2012; Liu et al., 2016, 2010). Therefore, we hypothesised that CRC cell‐derived EVs (CRC‐EVs) stimulate NOD1 activation and chemokine production in macrophages, thereby promoting CRC cell migration, to facilitate LM.

In this study, our findings revealed that CDC42, a small Rho guanosine‐5′‐triphosphate (GTP)ase, was delivered by CRC‐EVs into macrophages, consequently activating NOD1 to trigger an inflammatory response, subsequently potentiating CRC cell migration. We also showed that EVs from the plasma of CRC‐LM patients significantly activated the NOD1 signalling pathway in human peripheral blood mononuclear cells (PBMCs), while elevated NOD1 expression in tumour tissues was consistently associated with poor prognosis in patients with CRC‐LM. These findings not only elucidated the interaction between CRC‐EVs and host immune cell NOD1 activation but also provided potential targets for the diagnosis and treatment of CRC‐LM.

## 2 METHODS

### 2.1 Cell lines and cell culture

The human colon carcinoma cell lines HT29 and HCT116, human normal colon fibroblast cell line CCD18Co, and murine fibroblast cell line L929 were purchased from American Type Culture Collection (ATCC, Manassas, VA, USA). The murine colon adenocarcinoma cell line MC38 was obtained from China Cell Resource Center of Peking Union Medical College; the human monocytic line THP‐1 and human embryonic kidney 293T (HEK‐293T) were donated by Professor Wanli Liu from Tsinghua University.

HT29 cells, HCT116 cells, CCD18Co cells, MC38 cells and HEK‐293T cells were cultured in Dulbecco's modified Eagle's medium (DMEM; Invitrogen, Carlsbad, CA, USA) supplemented with 10% (v/v) fetal bovine serum (FBS; Invitrogen) and 1% (v/v) penicillin/streptomycin (Invitrogen). THP‐1 cells were grown in RPMI‐1640(Invitrogen) supplemented with 10% (v/v) FBS, 1% (v/v) penicillin/streptomycin, and 0.05 mM 2‐mercaptoethanol (Invitrogen). THP‐1 monocytes (1 × 10^6^) were maintained in 50 ng/ml phorbol 12‐myristate 13‐acetate (PMA; InvivoGen, San Diego, CA, USA) for 48 h, followed by incubation in RPMI‐1640 for another 24 h to allow differentiation. L929 cells were cultured in DMEM supplemented with 10% (v/v) FBS and 1 mM sodium pyruvate (Invitrogen) for three days to obtain the L929 cell culture supernatant. Murine bone marrow cells were isolated from C57BL/6J mice (6‐to 8‐week‐old) and cultured in DMEM supplemented with 20% (v/v) FBS and 20% (v/v) L929 cell culture supernatant for 7 days to generate bone‐marrow‐derived macrophages (BMDMs), as previously described (Weischenfeldt & Porse, 2008). All cells were maintained in a humidified incubator with 5% CO_2_ at 4°C. All cell lines were tested for mycoplasma contamination using a mycoplasma kit (M&C Gene Technology, Beijing, China), according to the manufacturer's protocols.

### 2.2 Tissue and blood sample collection

Human peripheral blood samples were obtained from healthy donors or CRC‐LM patients at the Tsinghua University Hospital or Peking University Cancer Hospital. Primary tumour and adjacent normal colon tissues, as well as metastatic liver and adjacent normal liver tissues, were obtained from CRC‐LM patients; primary tumour and adjacent normal colon tissues were obtained from CRC patients without LM (CRC‐NLM) at the Peking University Cancer Hospital and all cases were pathologically confirmed. All individuals provided informed consent according to the medical criteria of the institutional review board of Tsinghua University (Project No: 20200054). Plasma was harvested from peripheral blood and centrifuged at 400 × *g* for 10 min, aliquoted into 1 ml vials, and stored at –80°C for further analysis. PBMCs were isolated from peripheral blood with Ficoll‐PaquePLUS (density 1.077 g/ml, GE Healthcare, Chicago, IL, USA) as previously described (Wang et al., 2017) and immediately used for experiments.

### 2.3 Isolation of EVs

FBS were centrifuged at 100,000 ×*g* for 16 h at 4°C followed by filtering with a 0.22‐μm pore filter (syringe filter; 6786‐1302, GE Healthcare) to prepare ‘EV‐depleted FBS’ (Thery et al., 2006); the cells were cultured in DMEM with 5% ‘EV‐depleted FBS’ for 3 days until 80%–90% confluence. EVs were purified from the supernatant as previously described (Melo et al., 2015; Zhang et al., 2018). In brief, the harvested cell culture medium was centrifuged at 800 × *g* for 5 min, followed by centrifugation at 2000 × *g* for 10 min to remove cellular debris. The supernatant was filtered using a 0.22‐μm pore filter (syringe filter; 6786‐1302, GE Healthcare) and ultracentrifuged at 100,000 × *g* for 90 min at 4°C. The supernatant was discarded and the pellets were washed using phosphate‐buffered saline (PBS) before ultracentrifugation at 100,000 × *g* for 90 min at 4°C. The obtained pellets were resuspended in PBS.

EVs were purified from the human plasma samples using sise exclusion chromatography with commercially available Exo‐Spin midi columns (Cell Guidance Systems, St. Louis, MO, USA), following the manufacturer's protocol with slight modifications (Fuhrmann et al., 2018). Briefly, plasma (1 ml) was thawed on ice and centrifuged at 6000 × *g* for 10 min followed by 0.22‐μm filtration. The supernatant was further purified using the provided Exo‐spin columns, and the eluate containing the EVs was collected and concentrated using Amicon Ultra‐15 centrifugal units (molecular weight cut‐off 100 kD, Millipore, MA, USA) with centrifugation at 3,000 × *g* for 5 min to a final volume of 500–1000 μl.

The EV concentration was measured with a bicinchoninic acid (BCA) protein assay kit (Pierce). The EVs were then aliquoted into 20 μg vials and stored at –80°C for further analysis. EVs were diluted in PBS (1:100) and used for transmission electron microscopy (TEM) and nanoparticle tracking analysis (NTA) (details are described in the Supplementary Methods).

### 2.4 Gene expression analysis

RNA was isolated from cells and tissues using TRIzol Reagent (Life Technologies, Carlsbad, CA, USA). Total RNA (1 μg) was reverse‐transcribed in a 20 μl reaction system using the High‐Capacity cDNA Reverse Transcription Kit (Life Technologies). cDNA was diluted to 40 μl with nuclease‐free H_2_O (1:1), and 1 μl of the diluted cDNA was used to determine the mRNA expression by quantitative polymerase chain reaction (qPCR) analysis as previously described (Melo et al., 2015). The expression of cellular *IL‐6*, *TNF‐*α*, CCL1* and *CCL2* was normalised to that of *GAPDH;* the relative expression was calculated using the 2^–ΔΔCT^ method, as previously reported (Livak & Schmittgen, 2001). The CT value of NOD1 in tumour tissues and adjacent normal tissues was normalised to the average CT value of four housekeeping genes (*COPE*, *C1orf43, ENSA* and *GAPDH*) (Kasprzak et al., 2018; Xu et al., 2019). The primers for qPCR analysis are listed in Table S1.

### 2.5 Western blotting

Cells or EVs were lysed in RIPA lysis buffer (Beyotime Biotechnology, Suzhou, China) containing PMSF (Beyotime Biotechnology) and phosphatase inhibitor cocktail 3 (Sigma‐Aldrich). The protein concentration of each sample was measured using a BCA protein assay kit (Pierce). Equal amounts of the protein samples (20 μg per sample) were separated on an 8% or 10% SDS‐polyacrylamide gel; they were transferred onto an immun‐Blot PVDF membrane (Bio‐Rad, Hercules, CA, USA). After blocking the membrane with 5% skim milk in TBS with 0.1% Tween‐20, the blots were incubated with primary antibodies overnight at 4°C and probed with appropriate secondary antibodies conjugated with horseradish peroxidase. The protein bands were developed using chemiluminescence (#1863047, Thermo Scientific, Rockford, IL, USA). The antibodies used in this study are summarised in Table S2.

### 2.6 Tandem mass tag labelling quantitative proteomics analysis

Tandem mass tag (TMT)‐based quantitative liquid chromatography‐mass spectrometry (LC‐MS) analyses of EVs were performed at Tsinghua University Proteomics Resource Center. EVs were lysed in 8 M urea (pH = 8.0 in PBS) with 1 mM PMSF, 1 mM proteinase inhibitor cocktail 3 (Sigma‐Aldrich, Missouri, MO, USA), and the protein concentration of each sample was measured using a BCA kit (Thermo Scientific, Waltham, MA, USA) according to the manufacturer's instructions. The protein solution (50 μg) of each sample was reduced, alkylated, acetone‐precipitated, resuspended, digested, desalted, and labelled with TMT (Thermo Scientific); LC‐MS/MS analysis was performed as previously described (Yi et al., 2020). In brief, the raw MS data to identify and quantify peptides and proteins were analysed using the Proteome Discoverer 2.1 software. MS/MS spectra were searched against the UniprotKB *Homo sapiens* database (released on October, 2017, containing 20,168 entries). The parameters applied for database searching were as follows: enzyme specificity was set to trypsin; two missed cleavage sites were permitted; fixed modifications of TMT 4‐plex on lysine or peptide N terminus and cysteine carbamidomethylation; variable modification of oxidation on methionine; precursor tolerance was set to 10 ppm, and fragment ion tolerance to 0.02 Da; at least two unique peptides were required to identify proteins; 1% FDR (false discovery rate) at peptide‐spectrum match (PSM) level.

### 2.7 Preparation of conditioned medium from macrophages

Macrophages were differentiated from THP‐1 cells (1 × 10^6^) or BMDMs (1.5 × 10^6^) as described under ‘Cell lines and cell culture’ and then cultured overnight under starvation conditions in 6‐well plates with FBS‐free medium (RPMI‐1640 or DMEM). Cells were subsequently incubated with 20 μg/ml of EVs for 24 h. The cultured medium was harvested and centrifuged at 300 × *g* for 5 min to discard the cells. Conditioned medium (CM) was obtained by mixing the macrophage‐primed medium with complete medium (v/v = 1:1).

### 2.8 Wound‐healing assay

Wound‐healing assays were performed as previously described (Shang et al., 2019). In brief, MC38 or HT29 cells were cultured to a confluence of 80% in 6‐well plates, and a 500‐μm wide wound was created using a pipette tip. Serum‐free medium was used to wash the wounds, and cells were cultured with 2 ml of CM, as described under ‘Preparation of conditioned medium from macrophages’. Images of scratches were captured using a microscope (Olympus, Tokyo, Japan), and wound closure distance was calculated using the ImageJ software. The migration rate and relative migration rate were calculated using the following formula: migration rate = (1 – b/a) × 100 (a, original wound width; b, wound closure distance); relative migration rate = T/C × 100 (T, migration rate of the experimental group; C, migration rate of the control group).

### 2.9 Transwell assay

The migratory capacity of CRC cells in response to the CM of EV‐treated macrophages was assessed using transwell chambers with polycarbonate membrane filters with 24‐well inserts (6.5‐mm diameter and 8‐mm pore size; Corning Life Sciences, Tewksbury, MA, USA). MC38 or HT29 cells were cultured in FBS‐free medium (DMEM) for starvation overnight. Cells were suspended in FBS‐free medium (DMEM); 200 μl of cell suspensions (5 × 10^5^ cells/ml of MC38, or 2 × 10^6^ cells/ml of HT29) were added to the upper chambers with 500 μl of different types of CM in the bottom chambers. Cells were incubated for 18 h (MC38) or 24 h (HT29); thereafter, cell inserts were fixed with 4% paraformaldehyde and stained with 0.1 % crystal violet (Solarbio Science & Technology, Beijing, China) according to the manufacturer's instructions. The migrated cells were imaged at 200 × magnification, and the average number of migrated cells per field was calculated (five objectives). The relative migration rate was calculated using the following formula: T/C × 100 (T, number of migrated cells in the experimental group; C, number of migrated cells in the control group).

### 2.10 Mice and tumour models

Wild‐type (WT) C57BL/6J and NOD1^–/–^ mice (on C57BL/6J strain background) were obtained from Nanjing Biomedical Research Institute of Nanjing University (Nanjing, China). All mice were housed in standard pathogen‐free conditions with a 12 h light/dark cycle and free access to food and water in the animal research centre laboratory of Tsinghua University. Animal experiments were performed in accordance with the NIH Guide for the Care and Use of Laboratory Animals, with the approval of the Scientific Investigation Board of Tsinghua University, Beijing (2017‐LG‐001).

Twenty micrograms of EV‐MC38 in 100 μl of PBS was intravenously injected via the tail vein into 4‐week‐old male mice (WT or NOD1^–/–^) every other day; PBS was used as the control. Twenty days later, mouse was anesthetised with tribromoethanol and LM was induced via intra‐splenic inoculation of MC38 cells (1 × 10^6^/100 μl PBS); 3 min after injection, a splenectomy was performed on each mouse and the wound was closed with Vetbond Tissue Adhesive (3 M, St Paul, Minnesota). Administration of EV‐MC38 continued until day 35, at which point liver tissues were harvested from euthanised mice and photographed. The total number of visible LM were counted and one lobe of the liver tissue was fixed in 4% paraformaldehyde and embedded in paraffin. Next, 5‐μm‐thick sections were prepared, stained with haematoxylin and eosin (H&E), and photographed using Pannoramic SCAN (3DHISTECH Ltd., Budapest, Hungary).

### 2.11 Statistical analysis

GraphPad Prism version 7.0 (GraphPad Software) and unpaired Student's *t‐*tests were used to analyse data with only two sets. Univariate analysis using the log‐rank test was conducted to visualise (Kaplan–Meier curves) and assess disease‐specific survival (time from diagnosis to cancer‐related death or last follow‐up) in the longitudinal cohort study of patients with CRC. Statistical significance was two‐tailed and set at <0.05.

All other materials and methods used in this study are described in the Supplementary information.

## 3 RESULTS

### 3.1 CRC‐EVs activate NOD1 signalling to induce inflammatory responses in macrophages

To investigate the potential of NOD1 to sense tumour cell‐derived EVs, we first determined whether NOD1 signalling is activated following treatment of macrophages with EVs from CRC cells. To this end, EVs from CRC cells (HT29 cells) and normal colon cells (CCD18Co cells) were isolated using ultracentrifugation (Figure S1a). TEM and NTA showed that EVs were composed of a lipid bilayer, approximately 100 nm in diameter (Figure S1b, c). Western blotting verified the presence of positive markers (CD9, CD63, flotillin1, ALIX and TSG101) and the negative marker (calnexin), to further confirm the identity of EVs (Figure S1d).

**FIGURE 1:**
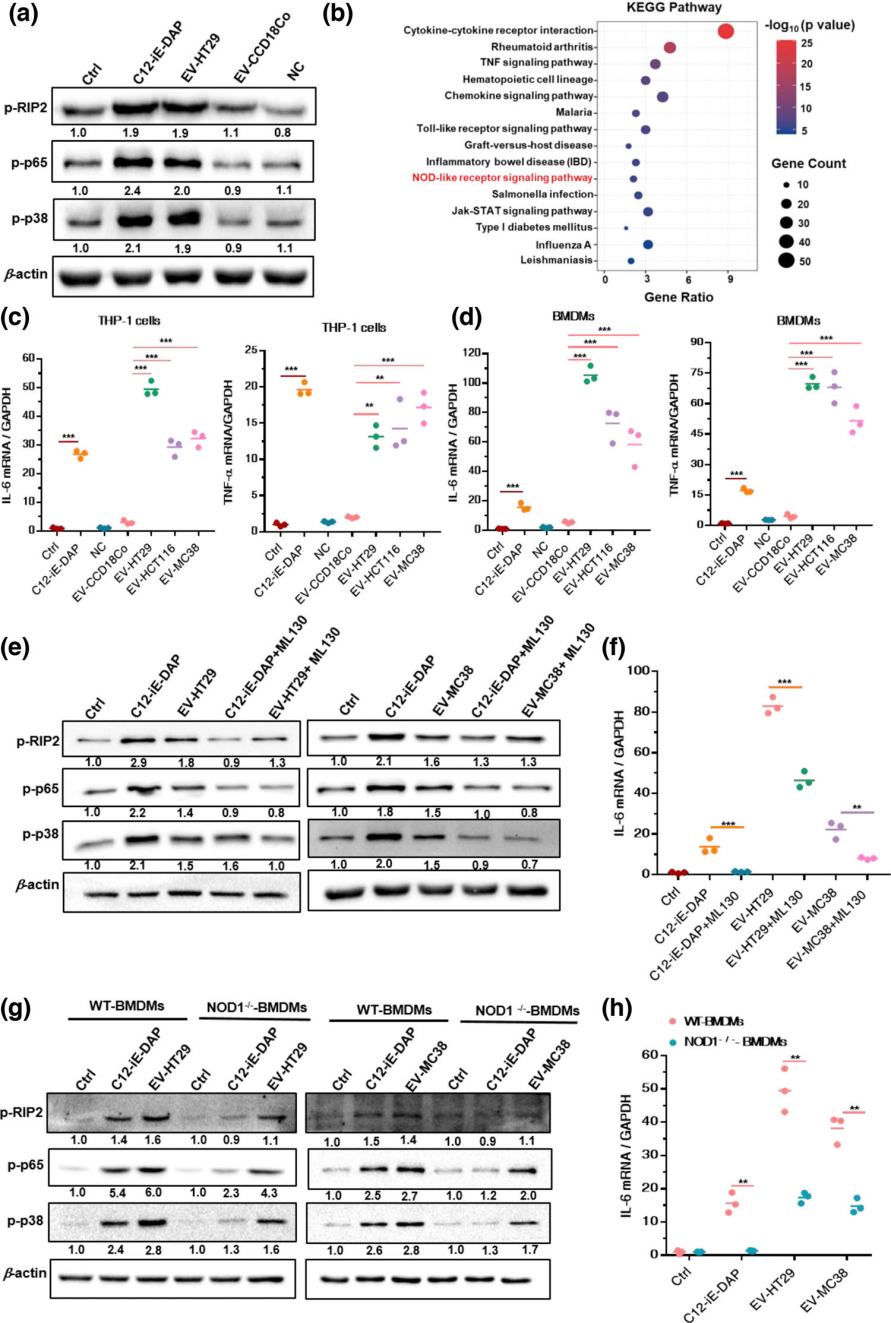
CRC‐EVs activate NOD1 signalling to induce inflammatory responses in macrophages. (a) Relative levels of p‐RIP2, p‐p65 and p‐p38 versus *β*‐actin. C12‐iE‐DAP represents the positive control; (*n* = 3). (b) KEGG pathway enrichment analysis for differentially expressed genes (|log_2_FC| ≥ 1, *p*‐value < 0.05). (c and d) Relative *IL‐6* and *TNF‐*α mRNA levels. C12‐iE‐DAP represents the positive control; (*n* = 3). (e and f) THP‐1 cells were pre‐treated with ML130 (20 μM) for 1 h and then treated with EV‐HT29 or EV‐MC38. (e) Relative levels of p‐RIP2, p‐p65 and p‐p38 versus *β*‐actin after 30 min incubation ;(*n* = 3). (f) Relative *IL‐6* mRNA expression levels after 90 min incubation ; (*n* = 3). (g and h) WT‐BMDMs and NOD1^–/–^‐BMDMs were treated with EV‐HT29 or EV‐MC38. (g) Relative levels of p‐RIP2, p‐p65 and p‐p38 versus *β*‐actin after 30 min incubation; (*n* = 3). (h) Relative *IL‐6* mRNA expression levels after 90 min incubation; (*n* = 3). NC: negative control; obtained from DMEM with 5% ‘EV‐depleted FBS’ with differential ultracentrifugation and 0.22‐μm filtration. Data are presented as the mean ± S.D. (error bars) of three independent experiments. *p < 0.05; **p < 0.01; ***p < 0.001. Student's *t*‐test was used to determine the significance level

Next, CRC cell‐derived EVs (EV‐HT29) and normal colon cell‐derived EVs (EV‐CCD18Co) were incubated with THP‐1 cells for 30 min, after which NOD1 signalling was detected. We found that EV‐HT29 markedly increased the levels of phosphorylated RIP2 (p‐RIP2), p65 (p‐p65) and p38 (p‐p38) in THP‐1 cells, compared with EV‐CCD18Co or negative control (NC; Figure 1a).

Additionally, the EV‐HT29‐activated and control THP‐1 cells were subjected to gene microarray analysis and Kyoto Encyclopedia of Genes and Genomes (KEGG) pathway enrichment analysis. Results showed that the NOD‐like receptor signalling pathway was significantly altered in the EV‐HT29‐primed THP‐1 cells (Figure 1b). Moreover, the proinflammatory cytokines interleukin 6 (IL‐6) and tumour necrosis factor‐alpha (TNF‐α), which are upregulated following NOD1 activation (Ma et al., 2020), were highly expressed in THP‐1 cells or BMDMs in the presence of different types of CRC‐EVs (EV‐HT29, EV‐HCT116, and EV‐MC38) but not EV‐CCD18Co (Figure 1c,d).

To confirm that RIP2 phosphorylation and downstream NF‐κB and p38‐MAPK activation are dependent on NOD1, a selective NOD1 antagonist (ML130) was used to block this protein in THP‐1 cells before CRC‐EV (EV‐HT29, EV‐MC38) treatment. Inhibition of NOD1 counteracted the increased levels of p‐RIP2, p‐p65, and p‐p38 in EV‐HT29‐ and EV‐MC38‐stimulated THP‐1 cells (Figure 1e). Moreover, in the presence of ML130 in THP‐1 cells stimulated with EV‐HT29 or EV‐MC38, a significant decrease was observed in the level of IL‐6, a marker of macrophage activation (Tanaka et al., 2016) (Figure 1f). Similar results were obtained when THP‐1 cells were treated with GSK583, a selective RIP2 inhibitor, to restrict activated NOD1 from triggering downstream signalling (Figure S2a, b).

To further verify the function of NOD1 in sensing CRC‐EVs, BMDMs from WT and NOD1^–/–^ mice were treated with EV‐HT29 or EV‐MC38 and NOD1 signalling was detected. As expected, the levels of p‐RIP2, p‐p65 and p‐p38 were markedly reduced in NOD1^–/–^‐ BMDMs stimulated with EV‐HT29 or EV‐MC38 compared to those in the WT‐BMDMs (Figure 1g). Significant suppression of *IL‐6* mRNA was also observed in CRC‐EV‐primed NOD1^–/–^‐BMDMs (Figure 1h).

Collectively, these data indicated that the NOD1 signalling pathway was specifically activated by CRC‐EVs, with consequent p‐RIP2‐mediated induction of NF‐κB and p38‐MAPK inflammatory signalling to stimulate IL‐6 secretion.

### 3.2 NOD1 activation by CRC‐EVs promotes in vitro CRC cell migration and in vivo colorectal LM

Given that tumour cell‐derived EV‐activated PRRs induce inflammatory responses to promote metastasis (Zhou et al., 2020) and NOD1 activation has been implicated in CRC metastasis (Jiang et al., 2020), we next explored whether CRC‐EV‐mediated NOD1 activation could promote CRC cell growth and migration.

**FIGURE 2:**
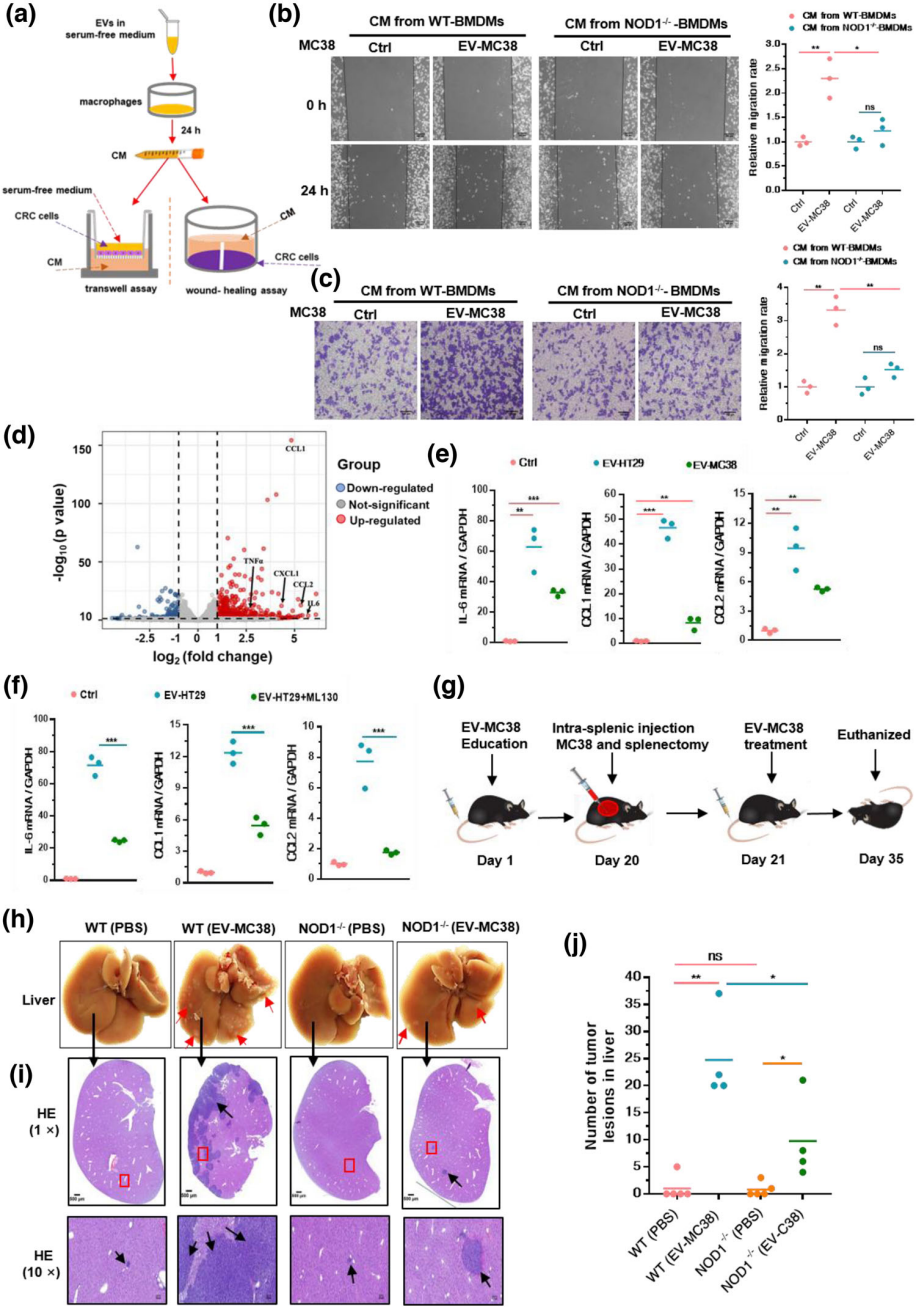
NOD1 activation by CRC‐EVs promotes in vitro CRC cell migration and in vivo colorectal liver metastasis. (a) Scheme of CM collected from EV‐activated macrophages in subsequent CRC cell migration analysis. (b) Wound‐healing and (c) transwell assays of MC38 cells in response to CM from WT‐BMDMs or NOD1^–/–^‐BMDMs incubated with EV‐MC38. Representative micrographs and relative migration rates are displayed; (*n* = 3). (d) Gene expression in THP‐1 cells of the EV‐HT29 group compared to that in the control group. Red dots, upregulated genes; blue dots, downregulated genes; grey dots, unchanged genes; (*n* = 3). (e) Relative *IL‐6*, *CCL1* and *CCL2* expression in THP‐1 cells incubated with EV‐HT29 or EV‐MC38; (*n* = 3). (f) Relative *IL‐6, CCL1* and *CCL2* levels in THP‐1 cells pre‐treated with ML130 (20 μM) and stimulated with EVs from HT29; (*n* = 3). (g) Schematic representation of the intra‐splenic colorectal metastasis model. EV‐MC38 (20 μg/100 μl of PBS) was injected intravenously (i.v.) into WT or NOD1^–/–^ mice every other day. Intra‐splenic inoculation of MC38 cells (1 × 10^6^/100 μl of PBS) and splenectomy was performed on day 20. Mice were euthanised on day 35. (h) Representative images of tumours in the liver. Red arrows indicate tumour lesions. Scale bar, 500 μm. (i) H&E staining of livers. Black arrows indicate tumour lesions. Scale bar, 50 μm. (j) Relative number of surface tumour lesions (*n* = 5, except WT (EV) and NOD1^–/–^ (EV), in which *n* = 4). Student's *t*‐test was used to determine the significance level; mean; error bar, S.E.M. *p < 0.05; **p < 0.01; ***p < 0.001

CM collected from macrophages incubated with CRC‐EVs was used to treat CRC cells (Figure 2a). CM from EV‐HT29‐activated THP‐1 cells or EV‐MC38‐activated WT‐BMDMs, but not NC, induced significant migration of HT29 and MC38 cells in the wound‐healing assay and transwell assays, respectively (Figure S3a‐d). Indeed, CM from EV‐MC38‐activated NOD1^–/–^‐BMDMs markedly suppressed MC38 cell migration compared to CM from EV‐MC38‐activated WT‐BMDMs in the wound‐healing assay (Figure 2b). CM from THP‐1 cells pre‐treated with ML130 to block NOD1 also prevented the augmentation of HT29 cell migration (Figure S4a). Meanwhile, transwell assays showed that CM from EV‐MC38‐primed NOD1^–/–^‐BMDMs and EV‐HT29‐primed THP‐1 cells treated with ML130 significantly attenuated the migration of MC38 and HT29 cells, respectively (Figure 2c, Figure S4b).

To evaluate whether proinflammatory cytokines or chemokines induced by CRC‐EV‐mediated NOD1 activation in macrophages contribute to the promotion of CRC cell migration, gene expression profiles of EV‐HT29‐activated and control THP‐1 cells were investigated. We analyzed the expression of proinflammatory cytokines (IL‐6) and chemokines (C‐C motif chemokine ligand 1(CCL1) and CCL2) that are known to contribute to cancer cell metastasis (De la Fuente Lopez et al., 2018; Olsen et al., 2017; Tanaka et al., 2016) and found that they were markedly upregulated in the EV‐HT29‐primed THP‐1 cells (68.7‐fold for *IL‐6*, 28.5‐fold for *CCL1* and 40.2‐fold for *CCL2*), according to the gene microarray analysis (Figure 2d, Figure S3c). Upregulation of *IL‐6, CCL1* and *CCL2* was confirmed in THP‐1 cells stimulated with EV‐HT29 or EV‐MC38 by qPCR analysis (Figure 2e). In addition, inhibition of NOD1 with ML130 counteracted the upregulation of *IL‐6, CCL1* and *CCL2* in EV‐HT29‐activated THP‐1 cells (Figure 2f). These observations indicated that macrophage NOD1 activation by CRC‐EVs induces inflammatory cytokine and chemokine production that potentiates CRC cell growth and migration.

Next, we investigated whether CRC‐EV‐activated NOD1 could promote CRC metastasis in vivo. EV‐MC38 or PBS (as NC) were injected into WT or NOD1^–/–^ mice via the tail vein every other day. As shown in Figure 2g, intra‐splenic inoculation of MC38 cells and splenectomy were performed to generate the LM model on day 20 after EV‐MC38 or PBS administration. We found that EV‐MC38 administration accelerated metastatic deposition in the liver compared to PBS administration in both WT and NOD1^–/–^ mice; however, more lesions developed in WT mice following EV‐MC38 administration than in NOD1^–/–^ mice (Figure 2h,j). Histologic analysis further demonstrated NOD1 deficiency markedly prevented migration and nodule formation in the liver following administration of EV‐MC38 (Figure 2i), suggesting that CRC‐EVs directly affect NOD1 signalling to promote LM in CRC. Taken together, these data suggested that NOD1 activation by CRC‐EVs is critical for inflammatory cytokine and chemokine production required for promoting CRC metastasis.

### 3.3 CDC42 in CRC‐EVs mediate NOD1 activation in macrophages

**FIGURE 3:**
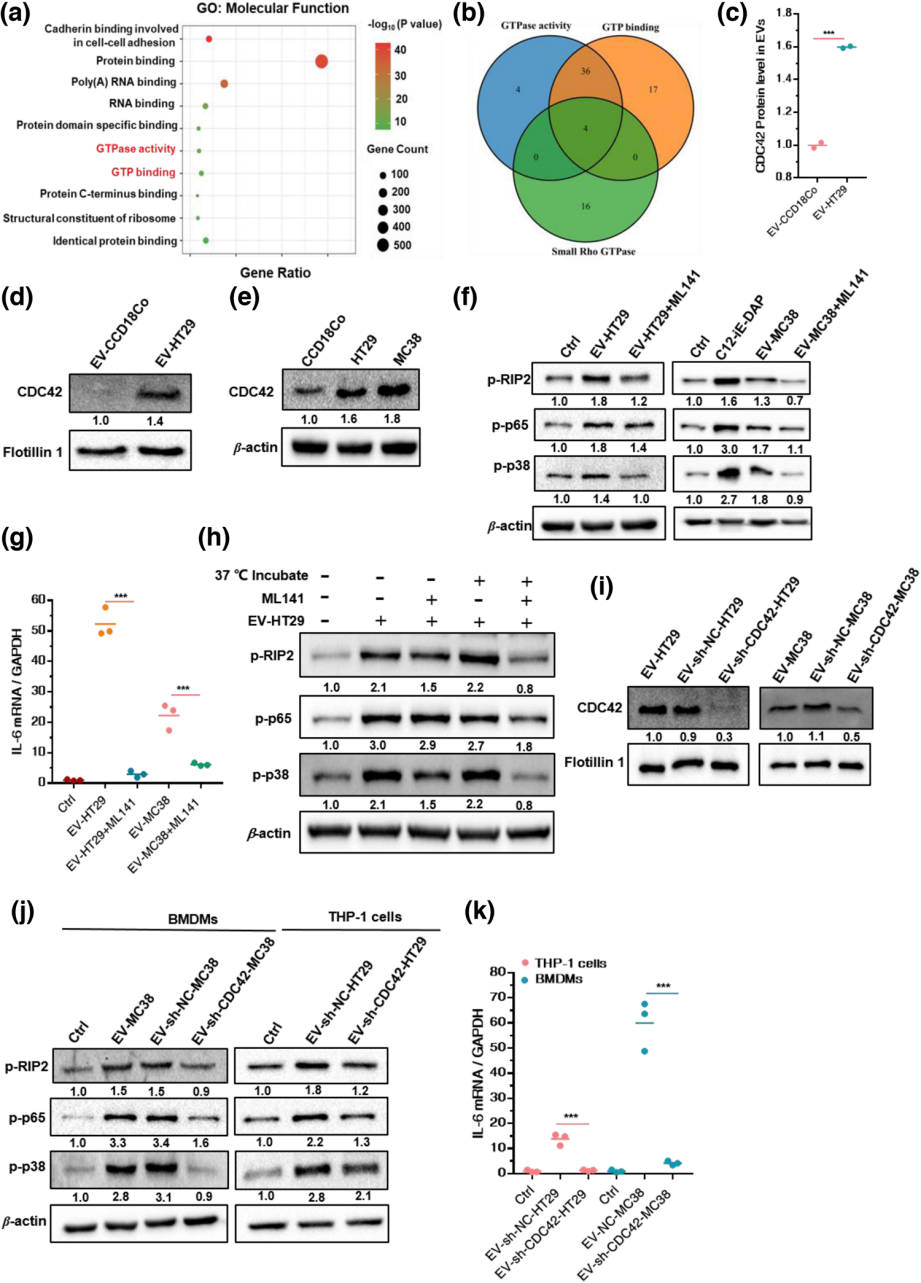
CDC42 in CRC‐EVs mediate NOD1 activation in macrophages. (a) Gene ontology (GO) analysis of proteins upregulated in EV‐HT29 compared to that in EV‐CCD18Co; only significantly enriched molecular functions are shown (*p*‐value < 0.05). (b) Overlap between known small Rho GTPases, proteins with molecular function of GTP binding, and GTPase activity. (c) Comparison of CDC42 protein levels in EV‐HT29 and EV‐CCD18Co; (*n* = 3). (d ane) Relative levels of CDC42 in (d) EVs (versus Flotillin 1) or cells (versus *β*‐actin); (*n* = 3). (f) Relative levels of p‐RIP2, p‐p65 and p‐p38 in THP‐1 cells following 30 min treatment with EV‐HT29 or EV‐MC38 pre‐incubated with ML141(20 μM); (*n* = 3). (g) Relative *IL‐6* expression in THP‐1 cells following 90 min treatment with EV‐HT29 or EV‐MC38 pre‐incubated with ML141 (20 μM); (*n* = 3). (h) Relative levels of p‐RIP2, p‐p6 and p‐p38 in THP‐1 cells treated for 30 min with EV‐HT29 (with or without pre‐incubation with ML141); (*n* = 3). (i) Relative level of CDC42 versus Flotillin 1 in EVs from *CDC42*‐knockdown CRC cells or NC cells; (*n* = 3). (j and k) THP‐1 cells or BMDMs were stimulated with EVs from CRC cells with CDC42 knockdown or the NC cells. (j) Relative levels of p‐RIP2, p‐p65 and p‐p38 in THP‐1 cells or BMDMs stimulated with EVs from *CDC42*‐knockdown CRC or NC cells; (*n* = 3). (k) Relative *IL‐6* expression in THP‐1 cells or BMDMs stimulated with EVs from *CDC42*‐knockdown CRC or NC cells; (*n* = 3). Data are presented as mean ± S.D. (error bars) of three independent experiments. *p < 0.05; **p < 0.01; ***p < 0.001. Student's *t*‐test was used to determine the significance level

We then sought to decipher how CRC‐EVs activate NOD1 in macrophages and their role in triggering inflammatory cytokine and chemokine release. EVs have been shown to contain diverse proteins and nucleic acids (Kowal et al., 2014). Given the role of NOD1 in the recognition of peptidoglycans (DAP) or effector proteins (SipA, SopE) present on pathogens (Keestra‐Gounder & Tsolis, 2017), liquid chromatography‐mass spectrometry (LC‐MS) was employed to analyse the protein cargo isolated from human CRC‐EVs (EV‐HT29) and the control (human normal colon cell line CCD18Co derived EVs, EV‐CCD18Co) (Figure S5a). We found that the abundance of 970 proteins significantly increased in EV‐HT29 compared to that in EV‐CCD18Co (a 1.2‐fold cut‐off was set to show biological significance) (data not shown). Bioinformatics analyses of gene ontology were performed on the significantly increased 970 proteins and revealed that GTP binding and GTPase activity were the enriched molecular functions (Figure 3a, Figure S5b, c).

Since small Rho GTPases have been reported to involve in NOD1 signalling activation (Keestra et al., 2013), we systematically analysed the proteins enriched in GTP binding and GTPase activity to identify small Rho GTPases (the proteins known as small Rho GTPases are shown in Table S4). We found that CDC42 was exclusively present in the overlap of GTP binding, GTPase activity and Rho GTPases (Figure 3b). Quantitative LC‐MS analysis further revealed that the level of CDC42 was significantly higher in EV‐HT29 than in EV‐CCD18Co (1.6‐fold; Figure 3c), as confirmed by immunoblotting analysis (Figure 3d). Additionally, CDC42 was abundantly expressed in HT29 and MC38 compared with that in CCD18Co (Figure 3e), indicating an increased level of CDC42 in CRC‐EVs originating from parent cells with higher levels of CDC42.

Next, we investigated whether CDC42 participates in CRC‐EV‐mediated activation of NOD1 signalling. ML141, a selective, non‐competitive and allosteric inhibitor of CDC42, locks the protein in an inactive conformation (Hong et al., 2013) and was used to block CDC42 activity. Pre‐incubation of ML141 with EV‐HT29 or EV‐MC38 significantly inhibited the upregulation of p‐RIP2, p‐p65 and p‐p38 expression as well as the increase in *IL‐6* mRNA expression in THP‐1 cells (Figure 3f,g), suggesting that inhibition of CDC42 in CRC‐EVs attenuated NOD1 activation. Indeed, without pre‐incubation of EV‐HT29, ML141 failed to exert an inhibitory effect on the upregulation of p‐RIP2, p‐p65 and p‐p38 expression in EV‐HT29‐activated THP‐1 cells (Figure 3h). Thus, CDC42 in CRC‐EVs was locked in an inactive form following pre‐treatment with ML141 before being transporting into macrophages, thereby, restricting its ability to trigger NOD1 signalling activation. Moreover, we found that ML141 could also inhibit the activation of NOD1 signalling and the release of IL‐6 induced by C12‐iE‐DAP (specific agonist of NOD1; Figure S6a,b), supporting the previous reports that Rho GTPases are necessary for NOD1 sensing of peptidoglycan fragments (Fukazawa et al., 2008; Keestra et al., 2013).

To further corroborate the role of CDC42 in NOD1 activation, we generated CDC42 knocked‐down HT29 and MC38 cells using short hairpin RNAs (shRNAs) (Figure S6c,d). CDC42 levels noticeably decreased in EVs derived from sh‐CDC42‐HT29 cells and sh‐CDC42‐MC38 cells compared to those in EVs from the corresponding NC cells (Figure 3i). The upregulated expression of p‐RIP2, p‐p65 and p‐p38 in EV‐sh‐NC‐MC38 activated BMDMs was similar to that in EV‐MC38‐activated BMDMs, while EV‐sh‐CDC42‐MC38, with deficiency of CDC42, failed to exert the same effect (Figure 3j). qPCR analysis also showed that CDC42 deficiency in EVs (EV‐sh‐CDC42‐MC38) downregulated *IL‐6* expression in BMDMs (Figure 3k). Similar results were obtained when THP‐1 cells were incubated with EV‐sh‐NC‐HT29 and EV‐sh‐CDC42‐HT29(Figure 3j,k). Thus, CDC42 in CRC‐EVs specifically mediated NOD1 activation in macrophages.

### 3.4 GTP‐CDC42 required for promotion of CRC cell migration by CRC‐EV‐activated NOD1 in macrophages

**FIGURE 4:**
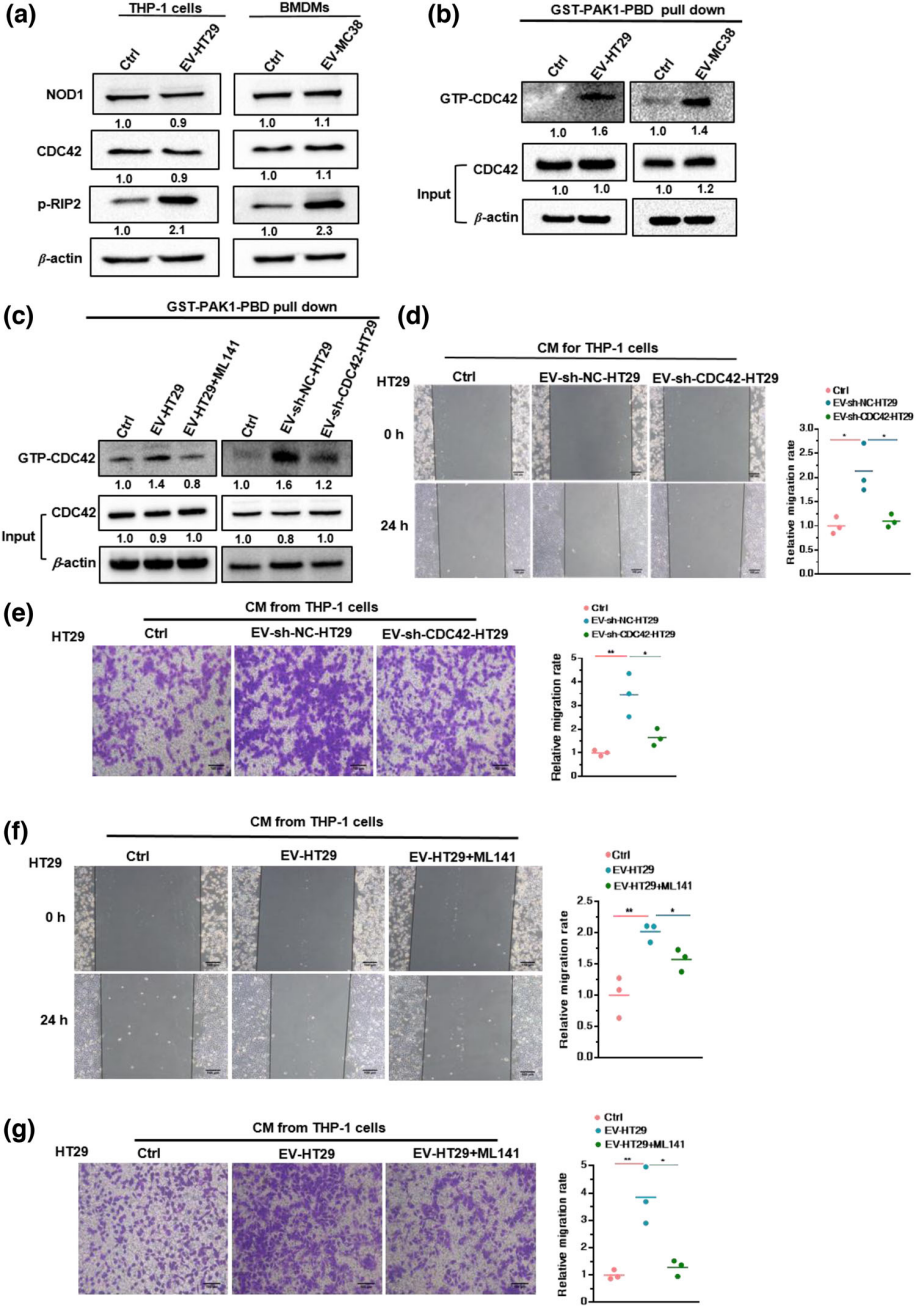
GTP‐CDC42 required for promotion of CRC cell migration by CRC‐EV‐activated NOD1 in macrophages. (a) Relative levels of NOD1, CDC42 and p‐RIP2 in THP‐1 cells incubated with EV‐HT29 (left), or BMDMs incubated with EV‐MC38 (right); (*n* = 3). (b) GTP‐bound state of CDC42 in THP‐1 cells incubated with EV‐HT29 or EV‐MC38; (*n* = 3). (c) GTP‐bound state of CDC42 in THP‐1 cells incubated with EV‐HT29 pre‐treated with ML141(20 μM; left), and THP‐1 cells incubated with EV‐sh‐NC‐HT29 or EV‐sh‐CDC42‐HT29 (right); (*n* = 3). (d) Wound‐healing assay and (e) transwell assay of HT29 cells in response to CM from THP‐1 cells stimulated by EV‐sh‐NC‐HT29 or EV‐sh‐CDC42‐HT29. (f) Wound‐healing assay and (g) transwell assay of HT29 cells following treatment with CM from THP‐1 cells incubated with EV‐HT29 pre‐incubated with ML141(20 μM). Representative micrographs and relative migration rates are displayed. Student's *t*‐test; error bar, S.E.M.*p < 0.05; **p < 0.01; ***p < 0.001

To elucidate the underlying mechanisms associated with activation of NOD1 by CDC42 in CRC‐EVs, we first examined the level of NOD1 and CDC42 in macrophages following CRC‐EV stimulation. However, NOD1 and CDC42 showed no obvious differences in EV‐HT29‐primed or EV‐MC38‐primed BMDMs compared with that in the control group (Figure 4a). CDC42, a GTPase, acts as a molecular switch between the inactive GDP‐bound state and the active GTP‐bound state (Jaffe & Hall, 2005). Therefore, we assessed the GTP‐bound CDC42 (GTP‐CDC42) in CRC‐EV stimulated THP‐1 cells via an active CDC42 pull‐down assay. The results showed that EV‐HT29 and EV‐MC38 could markedly increase the expression of GTP‐CDC42 (Figure 4b). Meanwhile, blockage of CDC42 via pre‐incubation of ML141 with EVs, or downregulation of CDC42 in EVs via shRNA, markedly diminished the upregulation of GTP‐CDC42 expression in THP‐1 cells (Figure 4c). These results suggest that the CDC42 cargo in CRC‐EVs was delivered into macrophages and, after adopting a GTP‐bound active state, mediated NOD1 activation.

Since CRC‐EVs activate NOD1 in macrophages to potentiate CRC cell migration (Figure 2), we postulated that the presence of CDC42 in CRC‐EVs is required for this process. Therefore, CM derived from THP‐1 cells stimulated with EV‐sh‐CDC42‐HT29 or EV‐sh‐NC‐HT29 was collected and incubated with HT29 cells. CDC42 deficiency in EVs clearly reduced the promoting effect of CM from EV‐primed THP‐1 cells on the migration of HT29 cells, as revealed by the wound‐healing assay (Figure 4d) and transwell assay (Figure 4e). Additionally, locking CDC42 in an inactive form via pre‐incubation with ML141 at 37°C for 1 h, also restricted the CM from the EV‐primed THP‐1 cells from promoting HT29 cell migration (Figure 4f,g). Collectively, these data indicated that GTP‐CDC42 is required for CRC‐EV‐induced NOD1 activation in macrophages to potentiate CRC cell migration.

### 3.5 EVs from CRC‐LM plasma mediate NOD1 activation in human PBMCs

**FIGURE 5:**
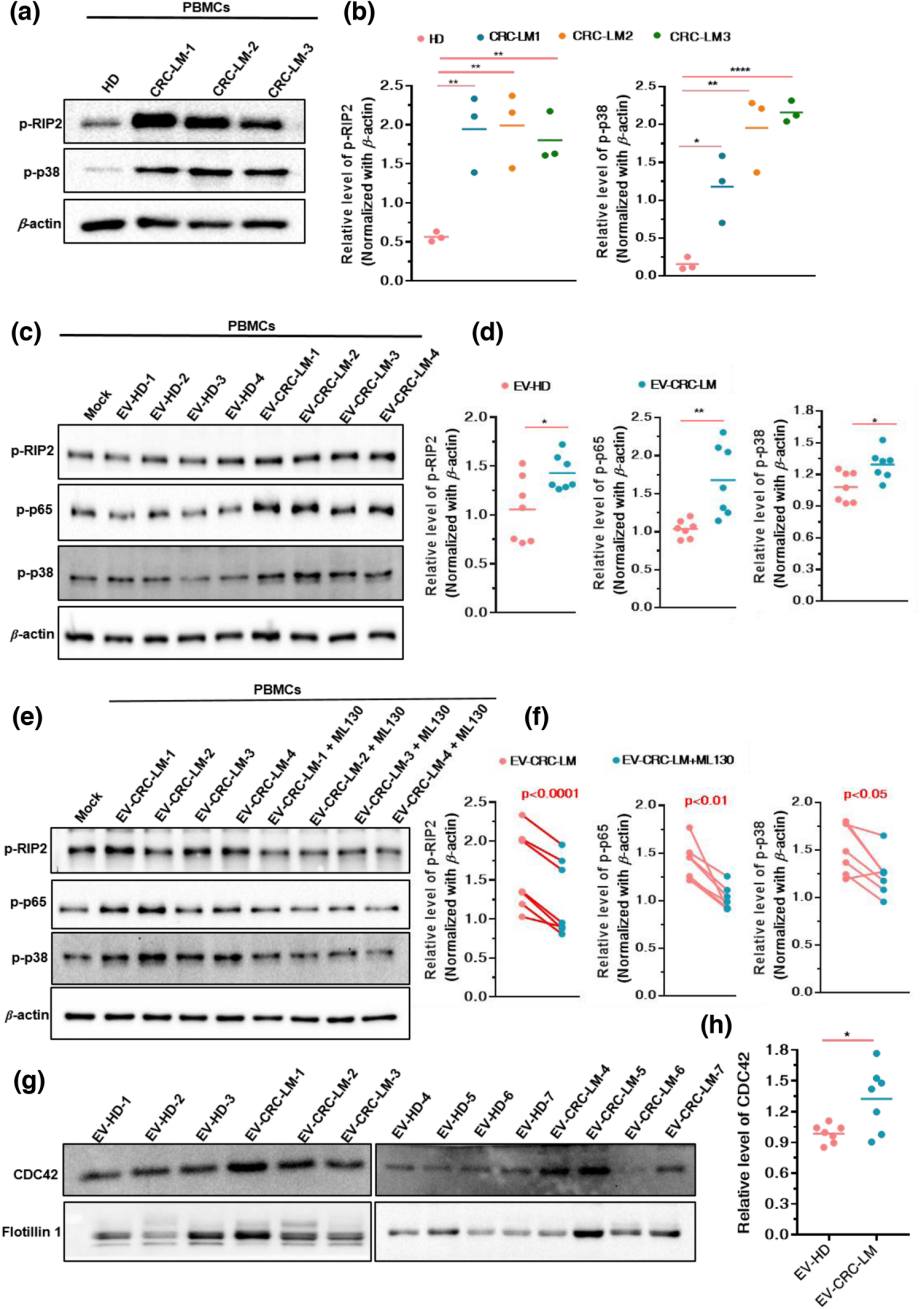
EVs from CRC plasma mediate NOD1 activation in human PBMCs. (a and b) Relative levels of p‐RIP2 and p‐p38 in PBMCs from healthy donors or patients with CRC‐LM detected using (a) western blotting and analysed by (b) Image J densitometric software; (*n* = 3). (c and d) Relative levels of p‐RIP2, p‐p65 and p‐p38 in PBMCs from healthy donors stimulated with EV‐HD (*n* = 7) or EV‐CRC‐LM (*n* = 7) detected using (c) western blotting and analysed by (d) Image J densitometric software. (e and f) Relative p‐RIP2, p‐p65 and p‐p38 abundance in PBMCs from healthy donors pre‐treated with ML130 (20 μM) and incubated with EV‐HD (*n* = 7) or EV‐CRC‐LM (*n* = 7) detected using (e) western blotting and analysed by (f) Image J densitometric software. (g) CDC42 and flotillin 1 expression in EV‐HD (*n* = 7) and EV‐CRC‐LM (*n* = 7). (h)The relative CDC42 protein expression in EV from HD and CRC‐LM assessed using Image J densitometric analysis and normalised based on the average of the HD densitometric values (*n* = 7). EV‐HD: EVs from healthy donors; EV‐CRC‐LM: EVs from patients with CRC‐LM. Mann–Whitney test was used to determine the significance level; mean; error bar, S.E.M. *p < 0.05; **p < 0.01; ***p < 0.001

To investigate the clinical relevance of NOD1 in the process of CRC‐LM, human PBMCs from healthy donors or patients with CRC‐LM were isolated and western blotting was performed to assess NOD1 signalling. As expected, the p‐RIP2 and p‐p38 levels were markedly increased in PBMCs from patients with CRC‐LM compared with those in PBMCs from healthy donors (Figure 5a,b), suggesting that activated NOD1 signalling had occurred in CRC‐LM.

Next, we explored whether EVs in the plasma induce the activation of NOD1 signalling in the PBMCs of patients with CRC‐LMs. EVs from the plasma donated by healthy donors or patients with CRC‐LMs were isolated using sise exclusion chromatography (Figure 7a) and characterised by NTA as well as via identification of CD9 and flotillin1 (Figure 7b,c). We found that EVs from the plasma of patients with CRC‐LM induced phosphorylation of RIP2, p65 and p38 in PBMCs, whereas EVs from the plasma of healthy donors did not (Figure 5c,d; Figure S7d). In addition, blockage of NOD1 by ML130 inhibited the increased in the levels of p‐RIP2, p‐p65 and p‐p38 mediated by EVs from the plasma of patients with CRC‐LM (Figure 5e,f; Figure S7e). We further detected a notably higher expression of CDC42 in the EVs from the plasma of patients with CRC‐LMs compared to that in the EVs from the plasma of healthy donors (Figure 5g,h). Therefore, EVs from the CRC‐LM plasma carried higher levels of CDC42 and activated NOD1 signalling in human PBMCs.

### 3.6 High NOD1 expression in tumour tissues promotes LM and poor prognosis in patients with CRC

**FIGURE 6:**
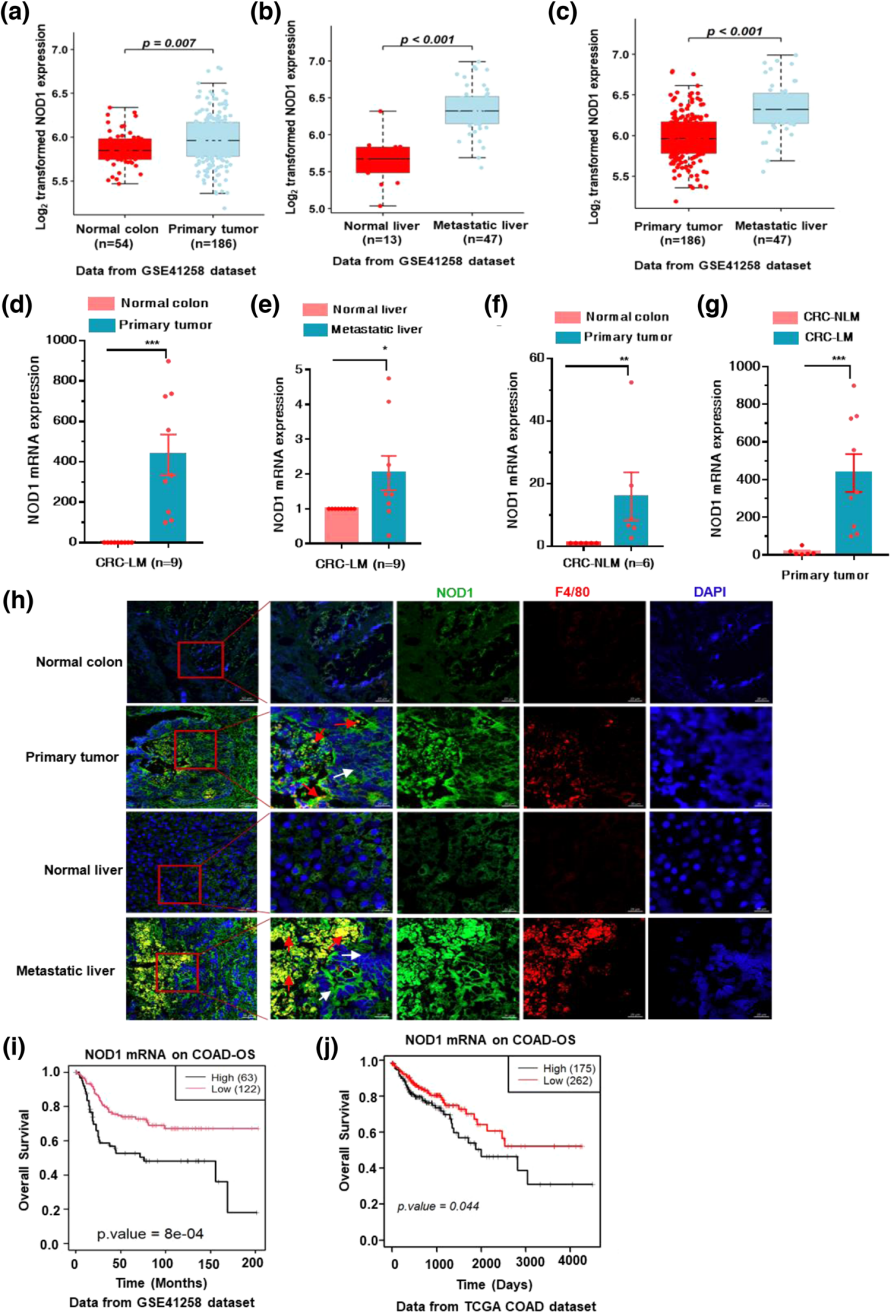
High NOD1 expression in tumour tissues promotes liver metastasis and poor prognosis in CRC patients. (a–c) Analysis of NOD1 expression in GEO dataset (ID: GSE41258). (a) normal colon tissues (*n* = 54) and primary tissues (*n* = 186); (b) normal liver tissues (*n* = 13) and metastatic liver tissues (*n* = 47); (c) primary tumour tissues (*n* = 186) and metastatic liver tissues (*n* = 47). (d and e) Relative *NOD1* levels in tumour tissues and adjacent normal tissues from patients with CRC‐LM. (d) Primary tissue and adjacent normal colon tissues (*n* = 9); (e) metastatic liver tissues and adjacent normal liver tissues (*n* = 9). (f) *NOD1* expression in primary tissue and adjacent normal colon tissues from non‐liver metastasis patients of CRC (CRC‐NLM) (*n* = 6). (g) Comparison of *NOD1* mRNA level in tumour tissues from CRC‐NLMs and primary tumour tissues from CRC‐LMs. (h) Representative fluorescent images of NOD1 and macrophage staining in tumour and normal tissues from CRC‐LM. NOD1, green; F4/80, red; DAPI, blue. Red arrow, macrophages; white arrow, tumour tissues. Scale bar, 500 μm, and 50 μm. (i and j) Relationship between overall survival of CRC patients and NOD1 expression in tumour‐adjacent tissues (*p* < 0.001, Kaplan–Meier test), (i) data from GEO dataset (ID: GSE41258); (j) data from TCGA. In (d)–(f), the CT values of NOD1 were normalised to the average CT value from four reference genes (*COPE, C1orf43, ENSA* and *GAPDH*). The *p*‐value in (a) to (g) was calculated using the nonparametric Mann–Whitney test. mean; error bar, S.E.M. *p < 0.05; **p < 0.01; ***p < 0.001

A previous study reported that colon tumours are associated with high NOD1 protein levels (Jiang et al., 2020). We exploited a Gene Expression Omnibus (GEO) dataset (GSE41258) microarray gene file from patients with CRC and CRC‐LM to investigate the role of NOD1 in primary CRC tumour tissues to promote LM. A significant upregulation of NOD1 expression was observed in primary tumour tissues and metastatic liver tissues compared to that in normal tissues (Figure 6a,b). Comparison of NOD1 levels between primary tumour tissues and metastatic liver tissues revealed that NOD1 is upregulated in the metastatic liver (Figure 6c). Consistent with results of the GSE41258 dataset analysis, NOD1 expression was markedly higher in primary tumour and metastatic liver than in the corresponding normal tissues, according to qPCR analysis of tissues from patients with CRC‐LM (Figure 6d,e).

Next, we quantified NOD1 expression in tumour tissues from CRC patients without liver metastasis (CRC‐NLM) and found that the level of NOD1 was also increased in tumour tissues (Figure 6f). Furthermore, comparison of NOD1 levels between primary tumour tissues from patients with CRC‐LM and tumour tissues from patients with CRC‐NLM, revealed that NOD1 upregulation was more pronounced in primary tumour tissues from patients with CRC‐LM (Figure 6g), implying that high levels of NOD1 may contribute to tumour metastasis in the liver.

Moreover, immunofluorescence analysis was conducted to observe NOD1 expression and macrophage infiltration in tumour and adjacent normal tissues from patients with CRC‐NLM. NOD1 expression was increased in primary tumour and metastatic liver tissues compared to that in paired adjacent normal tissues; the elevated expression of NOD1 was closely related to massive macrophage infiltration (Figure 6h). Considering that NOD1 was activated by EVs from CRC cells (Figure 1) and the plasma of CRC‐LM (Figure 5), these results implied that high levels of NOD1 in tumour‐associated macrophages may be activated by tumour‐derived EVs to induce inflammatory responses, subsequently promoting LM. Additionally, Kaplan–Meier analysis from both the GSE41258 dataset and Cancer Genome Atlas (TCGA) mRNA‐seq dataset revealed that higher NOD1 expression in tumours was associated with decreased overall survival (OS) rates (Figure 6i,j), suggesting a poor prognosis for patients with CRC and high levels of NOD1. Collectively, these findings indicated that high NOD1 expression in tumour tissues facilitates macrophage infiltration to promote metastasis in the liver and poor prognosis in CRC patients.

## 4 DISCUSSION

EVs serve as ‘messengers’ in intercellular communication through the delivery of their components (lipids, proteins or nucleic acids) into neighbouring or distant cells (Mathieu et al., 2019). EVs secreted by tumour cells could modify the extracellular matrix (ECM) or stromal cells to promote metastasis (Tkach & Thery, 2016). Previous studies have shown that multiple PRRs (TLR2, TLR3, TLR4, TLR7, TLR8 and RIG‐1) in stromal cells recognise tumour cell‐derived EVs and participate in tumour metastasis (Chalmin et al., 2010; Fabbri et al., 2012; Forte et al., 2012; Liu et al., 2016, 2010; Zhou et al., 2020). In this study, we revealed that a relatively new PRR, NOD1, was activated by CRC‐EVs, consequently inducing inflammatory responses in macrophages to promote tumour cell migration.

As an intracellular PRR, NOD1 senses bacterial‐derived muramyl peptides and participates in infections, autoimmune and inflammatory diseases (Caruso et al., 2014). Previous studies have revealed that NOD1 contributes to tumour development and progression, particularly in CRC and gastric cancer, which originate in sites with high host‐microbiome interactions (Jiang et al., 2020; Maisonneuve et al., 2021; Wang, 2012). Bacterial products activate NOD1 to promote CRC and increase CRC metastasis in the liver (Jiang et al., 2020; Maisonneuve et al., 2021). Here, we supported this concept that stimulation of NOD1 potentiates LM of CRC. Indeed, CRC‐EVs served as the stimulator.

CRC‐EVs were engulfed by macrophages (data not shown) and EV‐mediated production of proinflammatory cytokines and chemokines, such as IL‐6, CCL1 and CCL2, was dependent on NOD1, as confirmed by treatment with the selective NOD1 antagonist (ML130) and NOD1 knockout in macrophages. Recent evidence has indicted that *IL‐6* secreted by tumour‐associated macrophages enhances metastatic colonisation of CRC cells (Toyoshima et al., 2019; Zhong et al., 2020). Similarly, CCL1 and CCL2 have been shown to promote tumour metastasis by facilitating tumour‐associated macrophage recruitment (Chen et al., 2018, 2021). These data support our findings that CRC‐EV‐activated macrophages release IL‐6, CCL1 and CCL2 to potentiate CRC cell migration. In addition, this process originated from NOD1 stimulation by CRC‐EVs. Moreover, through quantitative LC‐MS, we found significant enrichment of CDC42, a small Rho GTPases, in CRC‐EVs, which was responsible for NOD1 activation. Studies have reported that active forms of small Rho GTPases are required for the activation of NOD1 signalling by peptidoglycan fragments (Keestra et al., 2013); and the excess CDC42 in EVs derived from TLR9‐activated macrophages is transported to naïve macrophages which further promoted cellular uptake of EVs (Zhang et al., 2019). In line with these reports, we demonstrated that the stimulation of NOD1 depended on the upregulation of the GTP‐bound active state of CDC42 in CRC‐EV‐activated macrophages. The high CDC42 cargo in CRC‐EVs was transported into macrophages, where it acquired its GTP‐bound active state and subsequently, mediated NOD1 activation, triggering the inflammatory response to promote tumour cell migration.

Activation of NOD1 facilitates CRC‐LM (Jiang et al., 2020). With CRISPR‐Cas9 technology to knockout NOD1 in mouse, we confirmed that NOD1 was critical for the promotion of CRC‐LM by CRC‐EVs. NOD1 deficiency markedly prevented migration and nodule formation in the liver following administration of CRC‐EVs. However, EV administration to NOD1‐deficient mice showed slight promotion of CRC‐LM. This could be attributed to the diverse components of EVs, which cause the activation of other factors to promote CRC‐LM, besides NOD1 activation. The enrichment of microRNA (such as miRNA‐21 (Shao et al., 2018), miRNA‐934 (Zhao et al., 2020), miRNA‐25, miRNA‐130 and miRNA‐425 (Wang et al., 2020)) in CRC‐EVs were correlated with CRC‐LM; they enhanced the M2 polarisation of macrophages to establish the inflammatory premetastatic niche. Indeed, miRNA‐25 in CRC‐EVs promotes CRC‐LM by inducing vascular permeability and angiogenesis (Zeng et al., 2018). The protein of ANGPTL1 could impede vascular leakiness to inhibit CRC‐LM; however, the level of ANGPTL1 is decreased in the EVs from CRC tumour tissues compared to that in normal tissues (Jiang et al., 2021). Therefore, CRC‐EVs promote CRC‐LM driven by the consolidated effects of different kinds of components in EVs. The enrichment of CDC42 to activate NOD1 was one of the critical factors.

NOD1 expression is upregulated in tumour tissues from all stages of CRC (Jiang et al., 2020). However, little is known regarding the correlations between NOD1 and CRC‐LM. We found that higher NOD1 expression was present in patients with LM, and was associated with massive macrophage infiltration, which facilitated tumour metastasis (Denardo & Ruffell, 2019). Moreover, CRC patients whose primary tumour tissues had higher NOD1 levels, showed poorer prognoses and increased risk of LM. Additionally, NOD1 signalling was activated in PBMCs of patients with CRC‐LM; the EVs from plasma with high CDC42 cargo accounted for this effect.

During analysis of NOD1 expression in tumour and normal tissues from patients with CRC, we, and others, found that a single reference gene was not sufficiently accurate to quantify target genes (Kasprzak et al., 2018, 2013). Several studies have demonstrated that many classical reference genes are dysregulated in tumours (Gur‐Dedeoglu et al., 2009; Xu et al., 2019). Thus, herein, more than 20 classical reference genes were analysed (data not shown), and four housekeeping genes (*COPE*, *C1orf43*, *ENSA* and *GAPDH*) with consistent expression in malignant and normal tissues, were confirmed to serve as effective normalisation standards to calculate *NOD1* mRNA expression. This investigation supports the notion that it is necessary to combine multiple housekeeping genes to accurately investigate dysregulated targets in tissue samples, and further provide alternative reference genes for CRC research.

**FIGURE 7:**
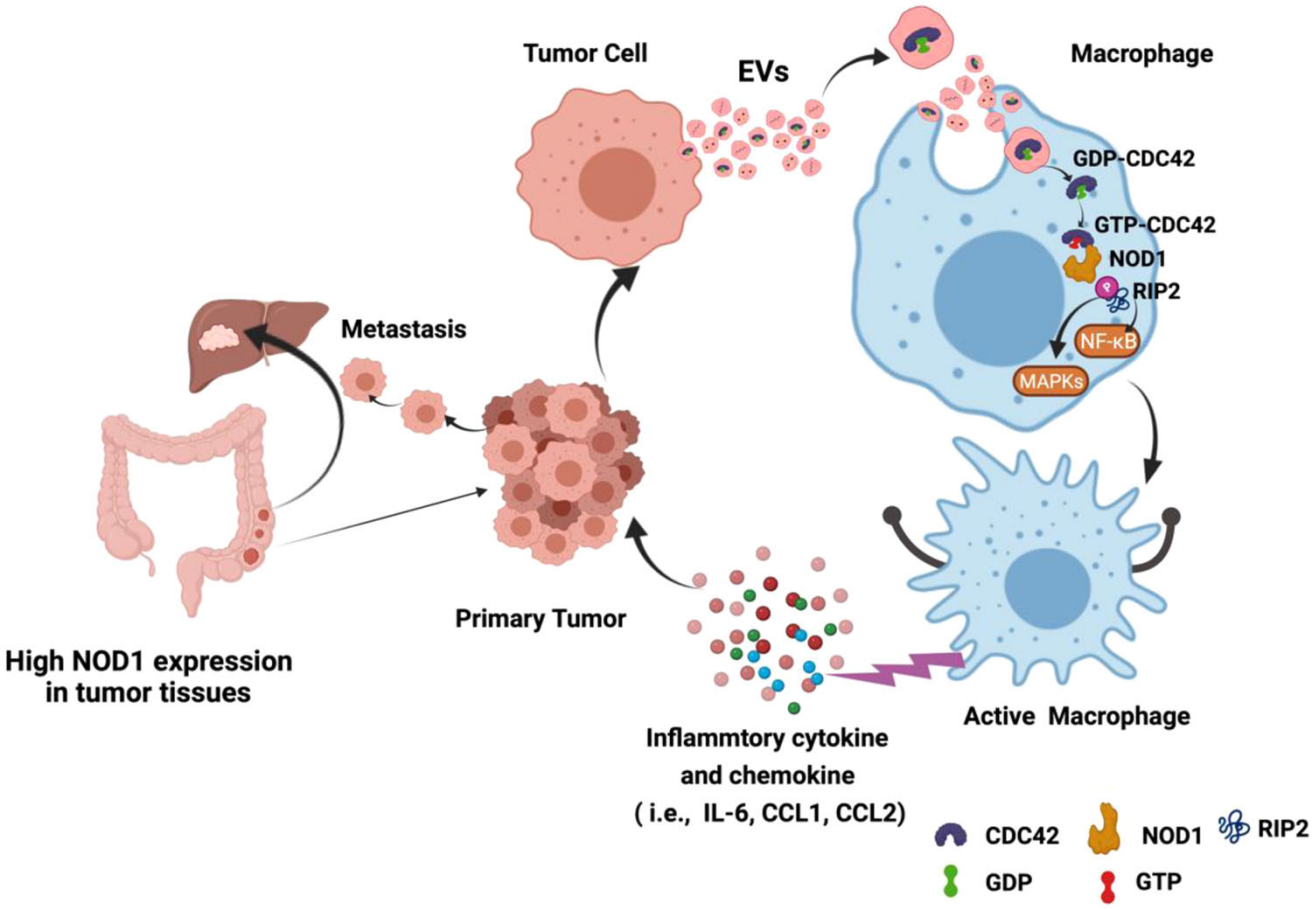
Proposed working model of CRC‐EV‐mediated NOD1 activation in promoting liver metastasis of CRC. EVs derived from CRC cells carry a high cargo of CDC42. When CRC‐EVs are transported into macrophages, CDC42 is delivered from CRC‐EVs to cells and switched to a GTP‐bound active state, which mediates NOD1 activation, with consequent RIP2 phosphorylation; it triggers downstream NF‐κB and p38‐MAPK‐dependent inflammatory cytokine and chemokine (i.e., IL‐6, CCL1 and CCL2) release, which promotes CRC metastasis

In summary, high CDC42 cargo levels in CRC‐EVs were delivered into macrophages, where they switched to a GTP‐bound active state to activate NOD1, with consequent RIP2 phosphorylation, triggering downstream NF‐κB and p38‐MAPK‐dependent inflammatory cytokine and chemokine (i.e., IL‐6, CCL1 and CCL2) release, which promoted CRC metastasis (Figure 7). However, whether activation of NOD1 by CRC‐EVs can induce macrophage M2 polarisation requires further investigation. Moreover, additional structural analysis is required to elucidate the interaction between CDC42 and NOD1, as well as the mechanism by which CDC42 in CRC cell‐derived EVs enters macrophages and switches from a GDP‐bound inactive state to a GTP‐bound active state. Collectively, these investigations will provide novel insights regarding the role of CDC42 in CRC‐EV activation of NOD1.

## AUTHOR CONTRIBUTIONS

Xiduan Wei, Yao Ma, Kun Wang and Gang Liu conceived and designed experiments. Xiduan Wei, Jingjia Ye, Yameng Pei, Jingyuan Tian and Guangxu Si performed biological experiments. Chunting Wang and Hongzhen Yang synthesised chemical reagents. Yao Ma, Kun Wang and Gang Liu proposed and supervised the study.

## CONFLICT OF INTEREST

The authors declare that they have no known competing financial interests or personal relationships that could have influenced the work reported in this paper.

## ETHIC STATEMENTS

Human sample study was conducted according to the medical criteria of the institutional review board of Tsinghua University (Project No: 20200054) and the protocol of biological sample library from Peking University Cancer Hospital. Animal experiments were performed in accordance with the NIH Guide for the Care and Use of Laboratory Animals, with the approval of the Scientific Investigation Board of Tsinghua University, Beijing (2017 ‐LG‐001).

## Supporting information

Supporting Information

## Data Availability

All relevant data are described within the paper and the Supplementary information.
