# Supplementary material for: Extracellular vesicles from colorectal cancer cells promote metastasis via the NOD1 signalling pathway
Source: J Extracell Vesicles. 2022 Sep 6;11(9):e12264. doi: 10.1002/jev2.12264 (PMC9448875; doi:10.1002/jev2.12264)

**Extracellular vesicles from colorectal cancer cells promote metastasis via the NOD1 signaling pathway**

Xiduan Wei, Jingjia Ye, Yameng Pei, Chunting Wang, Hongzhen Yang, Jingyuan Tian, Guangxu Si, Yao Ma^*^, Kun Wang^*^, Gang Liu^*^

**Supplemental Methods**

***Nanoparticle tracking analysis***

Nanoparticle tracking analysis (NTA) was used to detect the concentration and size distribution of EVs using NanoSight LM14 instrument (Malvern Instruments, UK) as previously described ^1^. In brief, EVs were suspended in PBS with the dilution of 1:100, and then injected to NanoSight LM14 instrument with constant flow. The trajectories of the particles were captured with a 60-second video for three times. Level 14 was set for the level of camera and the detection threshold was set at 4. Data were analyzed by NTA 3.1 software based on the captured videos.

***Transmission electron microscopy***

10 μl of EV-HT29 or EV-HCT116 (0.5 mg/ml) was loaded on a copper grid and then negatively stained with uranyl acetate solution for 5 min. The stained EVs were observed in Tecnai Spirit (120kV transmission electron microscopy) (FEI Company, Carlsbad, CA, USA).

***Generating the CDC42-knockdown cell lines***

Specific shRNA sequences in the human CDC42 gene (Gene ID: 998) and the mouse CDC42 gene (Gene ID: 12540) listed in Supplementary Table 3 were cloned into the pLKO.1 vector with puromycin resistance gene cassettes. An empty pLKO.1 vector was used as a negative control (NC). 293T cells were transfected with VSVG, Δ8.9, and specific CDC42 interfering lentiviral vectors or control vectors using LipofectamineTM 3000 Transfection Reagent (Thermo Fischer Scientific) according to the manufacturer’s protocols. The lentiviral particles in 293T cells cultured supernatant was harvested to infect HT29 or MC38 with 10 μg/ml polybrene (Beyotime, Jiangsu, China) for 24 h. The infected cells were selected using 2 μg/ml of puromycin (Invitrogen) resistance to generate stable cell lines.

***Active CDC42 pull-down assay***

Level of active CDC42 (GTP-CDC42) in macrophages were detected with the Active CDC42 Pull-Down and Detection Kit (Thermo Fischer Scientific) according to the manufacturer’s instructions.

***Microarray analysis***

Microarray analysis of cellular mRNA was performed at Annoroad Gennome Corporation (Beijing, China) using NextSeq 550AR (Illumina, USA). THP-1 cells were incubated with or without EV-HT29 for 24 h. Total RNA was isolated from cells and tissues using TRIzol^TM^ Reagent (Life Technologies, Carlsbad, CA, USA) and used for microarray analysis.

***Bioinformatics analyses***

The proteins from LC-MS analysis or Genes from microarray analysis were performed using an online resource of bioinformatics tools, DAVID (the Database for Annotation, Visualization and Integrated Discovery, https://david.ncifcrf.gov). The list of regulated proteins (fold change >1.2) or genes (|log2 fold change| ≥ 1, p-value < 0.05) were submitted to DAVID v6.8. to analyze the enrichment of gene ontology (GO) and Kyoto Encyclopedia of Genes and Genomes (KEGG) pathway.

***Tissue processing and immunofluorescence***

Primary tumor and adjacent normal colon tissues, metastatic liver and adjacent normal liver tissues were obtained from CRC-LM patients were dissected and embedded in Tissue-tek O.C.T. Then, 5-μm-thick O.C.T tissue cryosections were stained with primary antibodies and incubated with Alexa Fluor 488 or 647 secondary antibodies Fluorescent images were photographed using a confocal microscope (LSM980 Airyscan2, Zeiss) and analyzed with Imaris 7. The antibodies in this study are summarized in Supplementary Table 2.

Supplemental Reference

1. Zhang, Y.*, et al.* Extracellular vesicles derived from ODN-stimulated macrophages transfer and activate Cdc42 in recipient cells and thereby increase cellular permissiveness to EV uptake. *Sci Adv* **5**, eaav1564 (2019).

2. Narumiya, S. & Thumkeo, D. Rho signaling research: history, current status and future directions. *FEBS Lett* **592**, 1763-1776 (2018).

**Supplemental Figure legend**

**Supplementary Figure 1 Characterization of EVs purified from supernatant of cells. (a)** Schematic illustration of purified EVs from supernatant of cells cultures by ultracentrifugation. **(b)** Representative images of EV-HT29 or EV-HCT116 by TME. **(c)** NanoSight nanoparticle tracking analysis the size of EVs derived from cells cultures; (n = 3). **(d)** The protein levels of CD9, Flotillin1, CD63, ALIX, TSG101 and Calnexin in EVs and the donor cells were detected using western blotting; (n = 3).

**Supplementary Figure 2 CRC-EVs activate NOD1 signaling in macrophages. (a)** Relative *TNF-α* mRNA expression levels following pre-treatment of THP-1 cells with ML130 (20 μM) for 90 min; (n = 3). **(b)** Relative levels of p-RIP2, p-p65, and p-p38 versus β-actin following pre-treatment of THP-1 cells with GSK583 (1 μM) for 30 min；(n = 3). **(c)** Relative *IL-6* mRNA expression levels following pre-treatment of THP-1 cells with GSK583 (1 μM) for 90 min; (n = 3). **(d)** WT-BMDMs and NOD1^-/-^-BMDMs were treated with EV-HT29 or EV-MC38. Relative *TNF-α* expression levels after 90 min incubation; (n = 3). Data are presented as the mean ± S.D. (error bars) of three independent experiments. Student’s t-test was used to determine the significance level. *p < 0.05; **p < 0.01; ***p < 0.001.

**Supplementary Figure 3 Macrophages primed by CRC-EVs promote CRC cells migration. (a)** Wound-healing and **(b)** transwell assays of HT29 cells in response to CM from THP-1 cells incubated with EV-HT29 or NC. Representative micrographs and relative migration rates are displayed; (n = 3). **(c)** Wound-healing and **(d)** transwell assays of MC38 cells in response to CM from WT-BMDMs incubated with EV-MC38 or NC. Representative micrographs and relative migration rates are displayed; (n = 3). Student’s t-test was used to determine the significance level; mean; error bar, S.E.M. *p < 0.05; **p < 0.01; ***p < 0.001.

**Supplementary Figure 4 NOD1 activation by CRC-EVs in macrophages promotes CRC cells migration. (a-b)** THP-1 cells were pre-treated with NOD1 antagonist (ML130; 20μM) for 1 h, followed to treat with EV-HT29 for 24 h. **(a)** Wound-healing assays and **(b)** transwell assays of HT29 cells in response to different CMs from THP-1cells. Representative micrographs and the relative migration rates are displayed; (n = 3). Student’s t-test was used to determine the significance level; mean; error bar, S.E.M. *p < 0.05; **p < 0.01; ***p < 0.001.**(c)** Comparison of the relative *IL-6, TNF-α, CCL1, CCL2* mRNA levels in the EV-HT29 group and the control group quantified by gene microarray analysis.

**Supplementary Figure 5 Characterization protein cargo of EV-HT29 and EV-CCD18Co. (a)** Schematic illustration of LC-MS to analyze the protein cargo from CRC cells-derived EVs (EV-HT29) and normal colon cells-derived EVs (EV-CCD18Co). **(b)** Proteins increased in EVs-HT29 compared with EVs-CCD18Co (1.2-fold cutoff was chosen to show biological significance) were performed bioinformatics analyses of GO analysis. The significantly enriched cellular component and biological process were shown (p-value < 0.05).

**Supplementary Figure 6 CDC42 in CRC-EVs mediate NOD1 activation in macrophages. (a)** Relative levels of p-RIP2, p-p65, and p-p38 versus β-actin following treatment of THP-1 cells with ML141(20 μM) for 30 min; (n = 3). **(b)** Relative *IL-6* levels following treatment of THP-1 cells with ML141(20 μM) for 90 min; (n = 3). **(c-d)** Specific interfering lentiviral vectors containing puromycin resistance gene cassettes were used to establish shRNA-mediated knockdown CDC42 in HT29 cells and MC38 cells. Western blotting and qPCR analysis of the level of CDC42 in the stable CDC42 knocked-down cell lines and the NC cell lines; **(c)** sh-CDC42-HT29 or **(d)** sh-CDC42-MC38; (n = 3). *p <0.05; **p <0.01; ***p <0.001; unpaired Student’s t-test.

**Supplementary Figure 7 EVs from CRC plasma mediate NOD1 activation in human PBMCs. (a)** Schematic illustration of purified EVs from human plasma by size exclusion chromatography (SEC). EVs were enriched using Amicon Ultra-15 centrifugal units (molecular weight cut-off 100 kDa). **(b)** Nanoparticle tracking analysis the size of EVs derived from human plasma. **(c)** The protein levels of Flotillin 1, CD9 in EVs from healthy donors or CRC-LMs were detected using western blotting. **(d)** PBMCs from healthy donors were stimulated by EV-HD or EV-CRC-LM for 30 min. Samples were collected for immunoblotting analysis. **(e)** PBMCs from healthy donors were pre-treated with ML130 (20 μM) for 1 h, then stimulated by EV-CRC-LM for 30 min. Samples were collected for immunoblotting analysis.

**Supplemental Figure**

Supplementary Figure 1


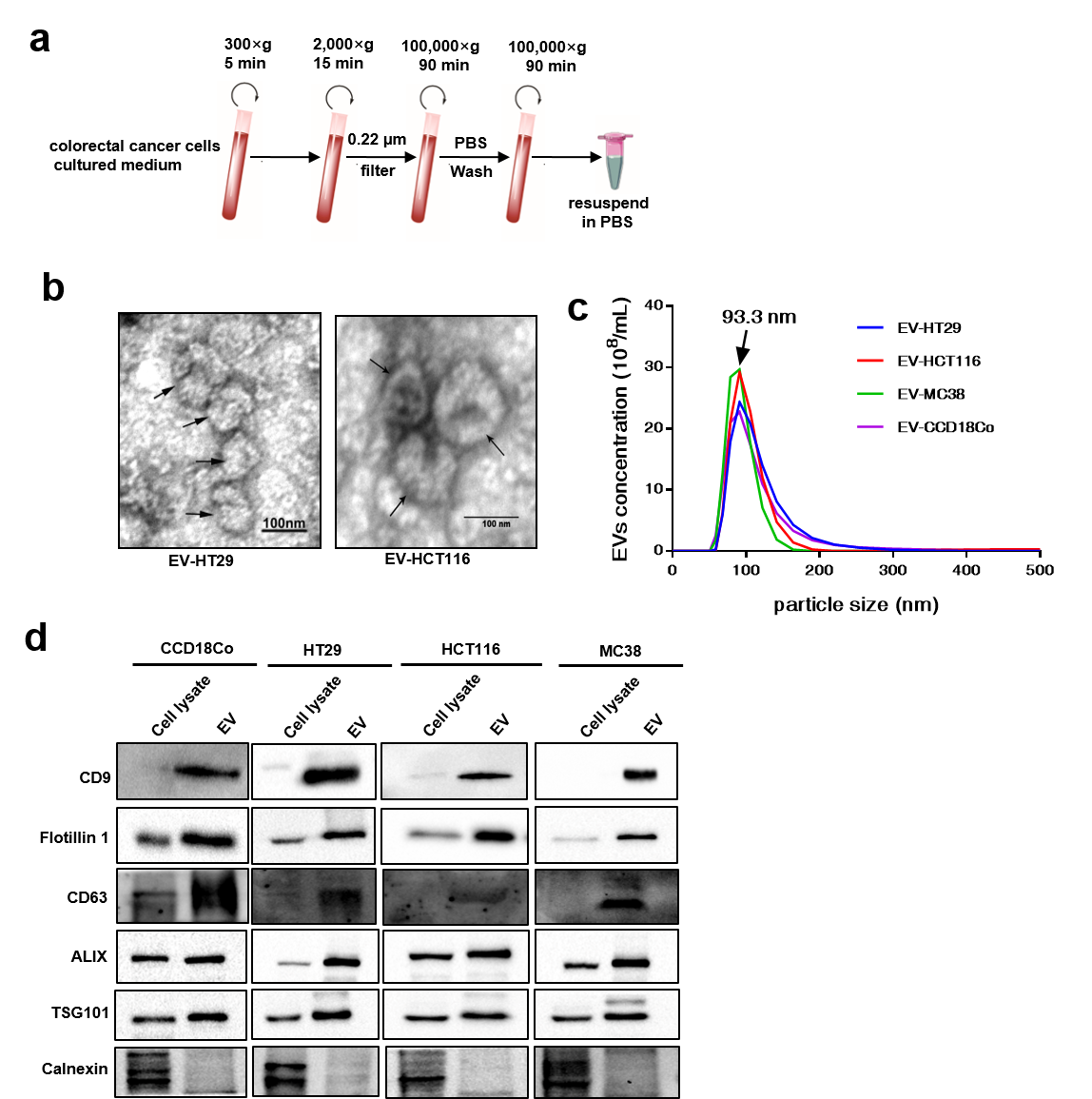


Supplementary Figure 2


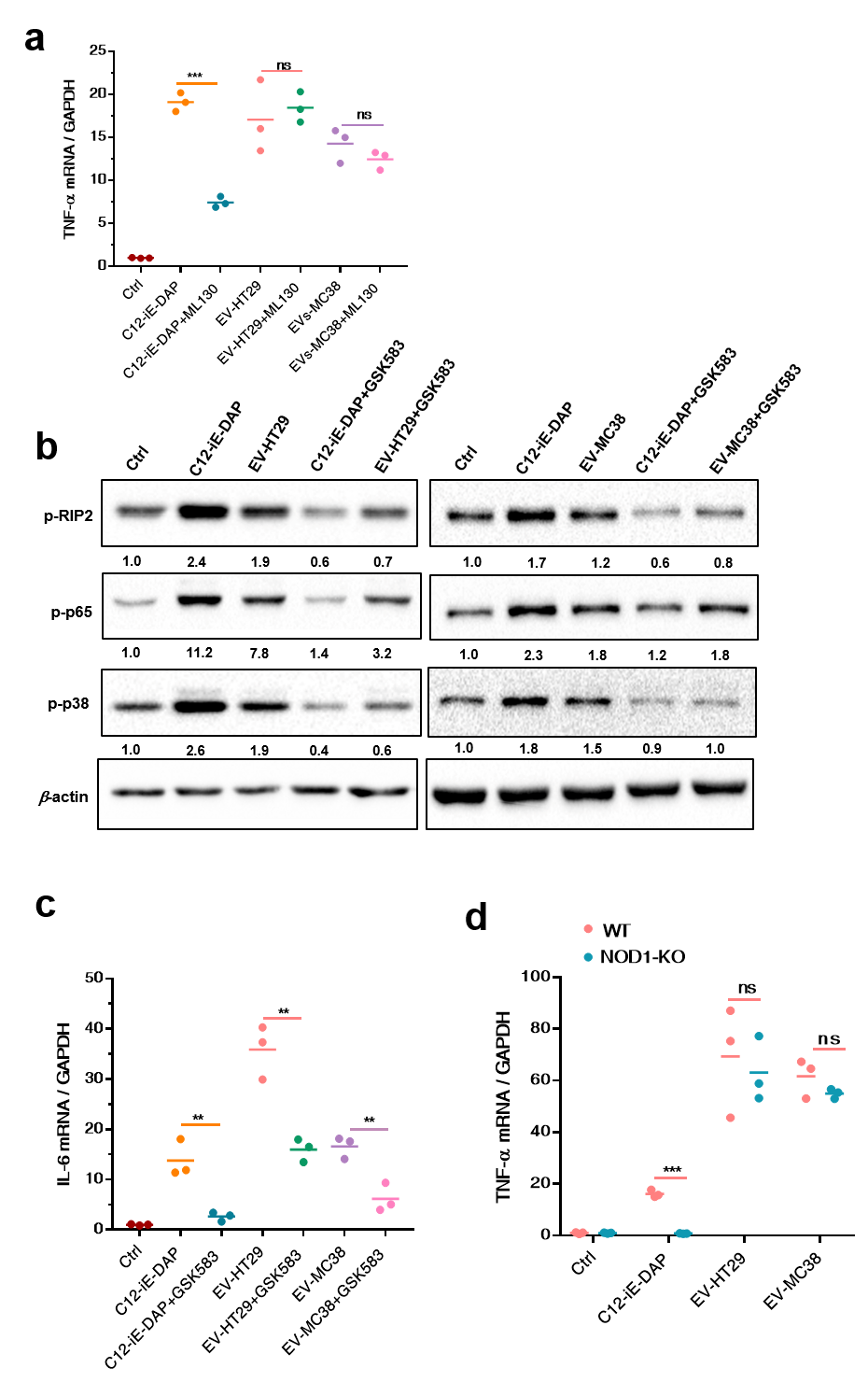


Supplementary Figure 3


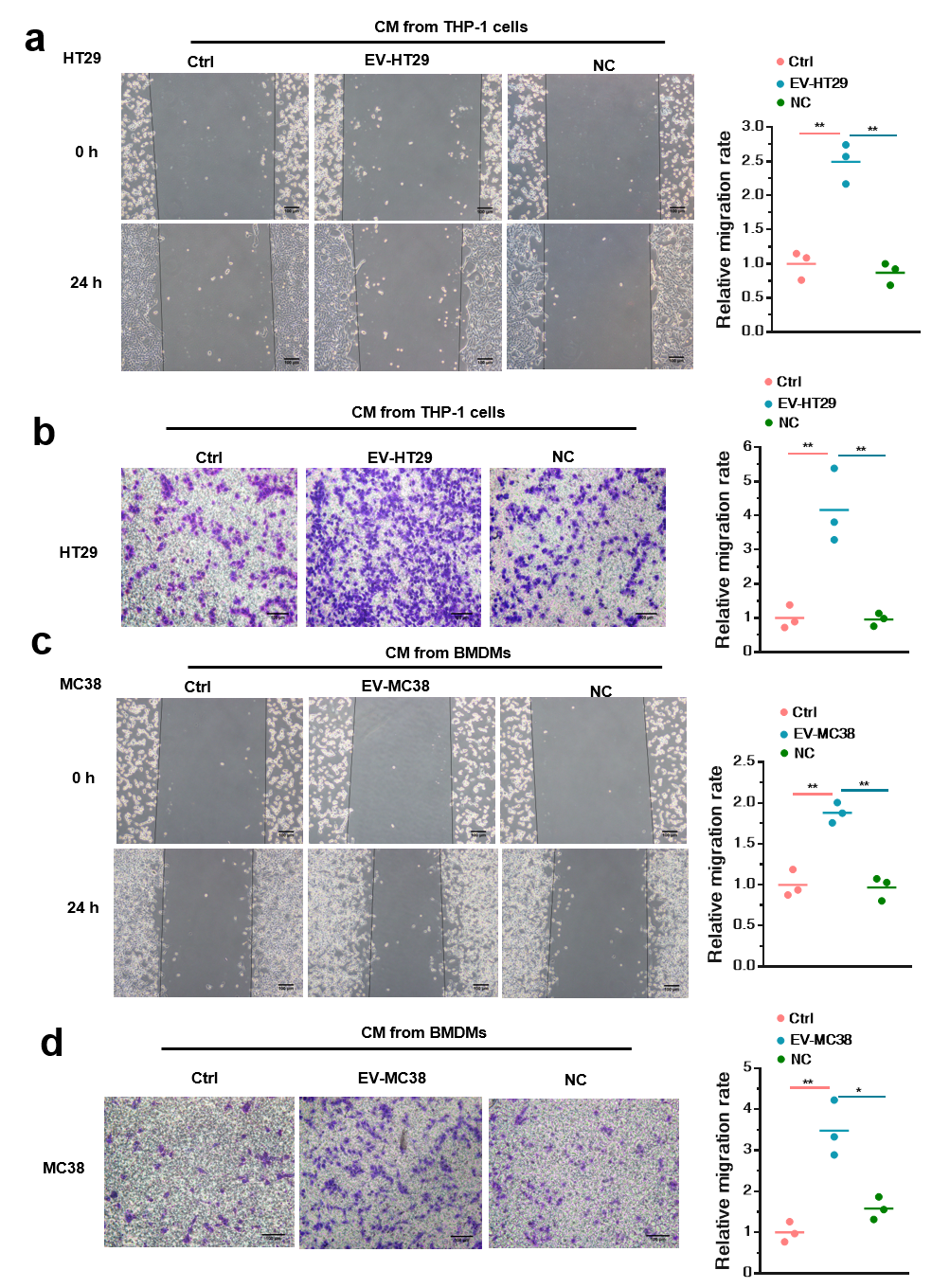


Supplementary Figure 4


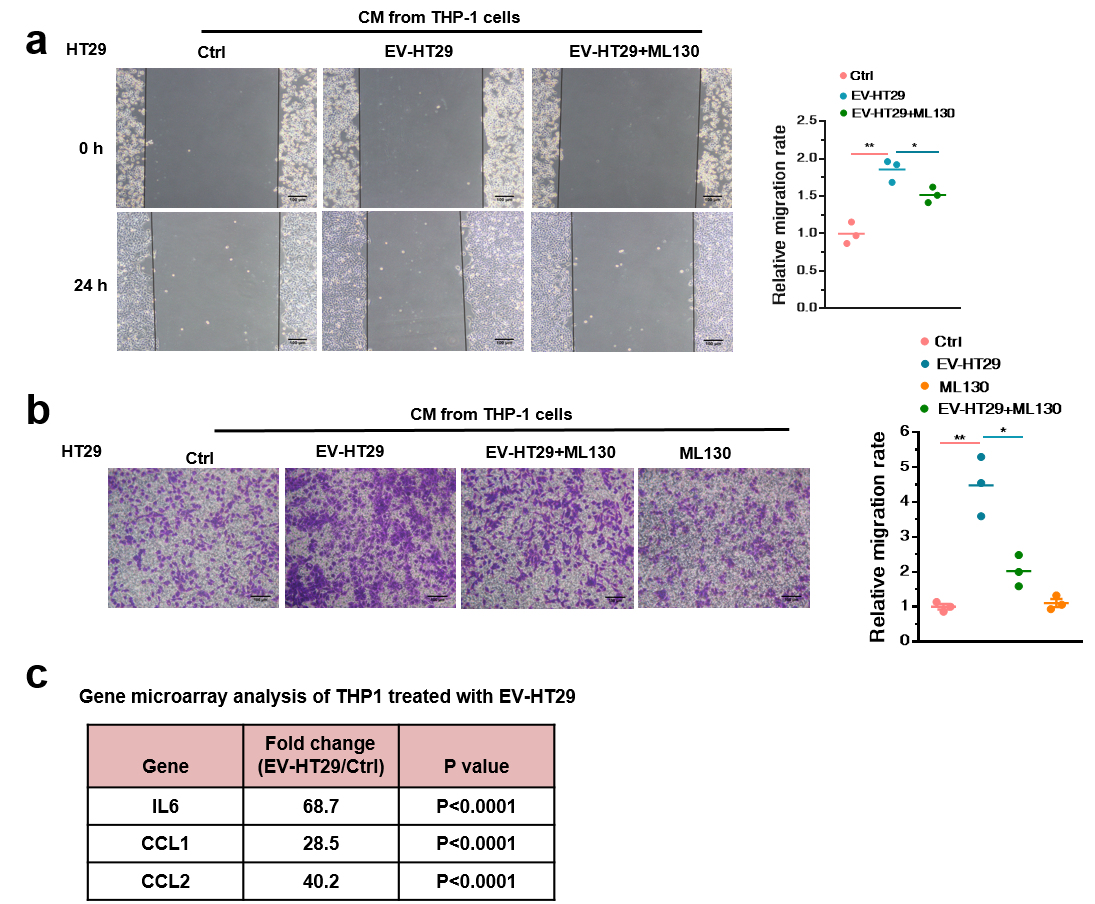


**Supplementary Figure 5**


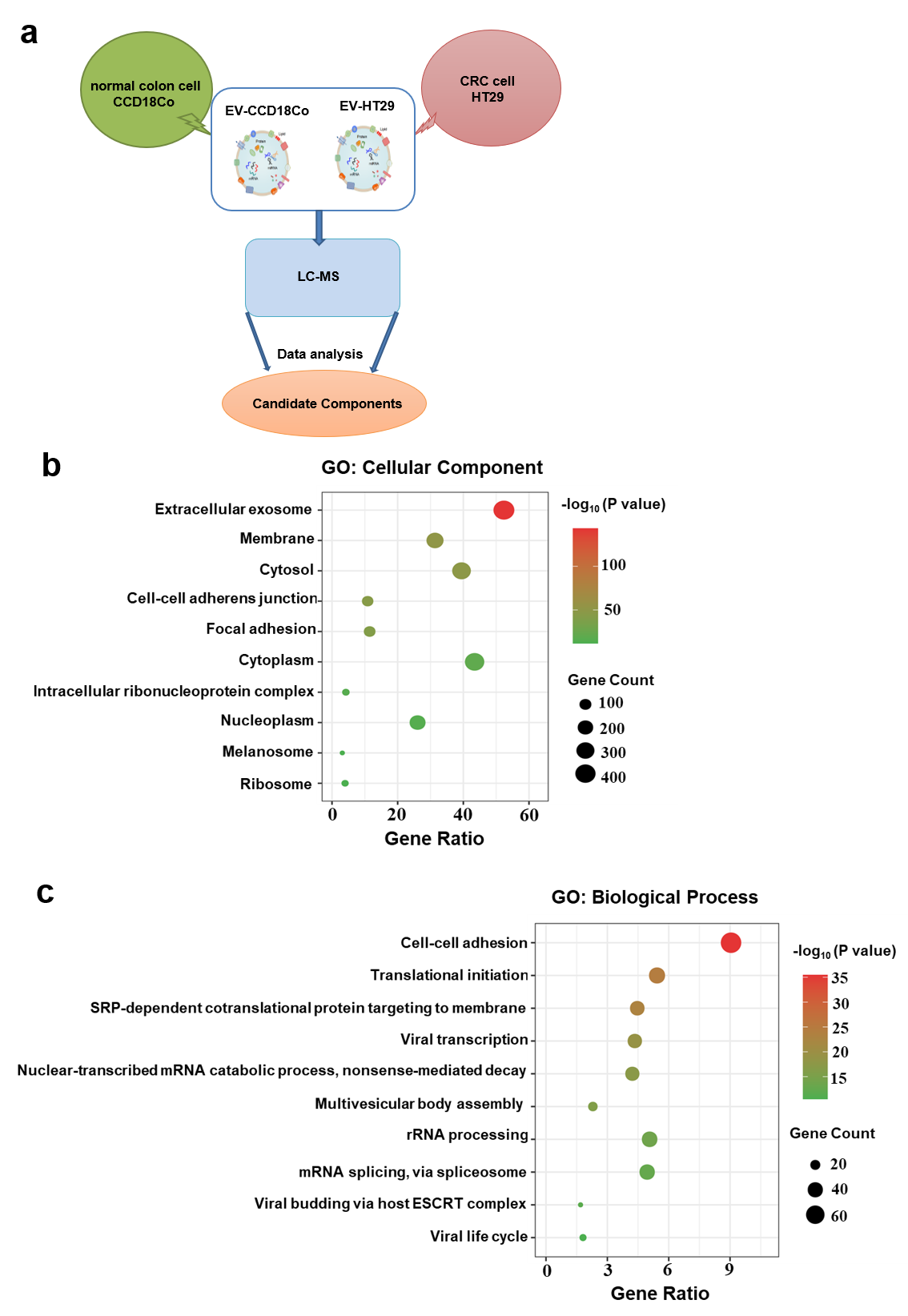


Supplementary Figure 6


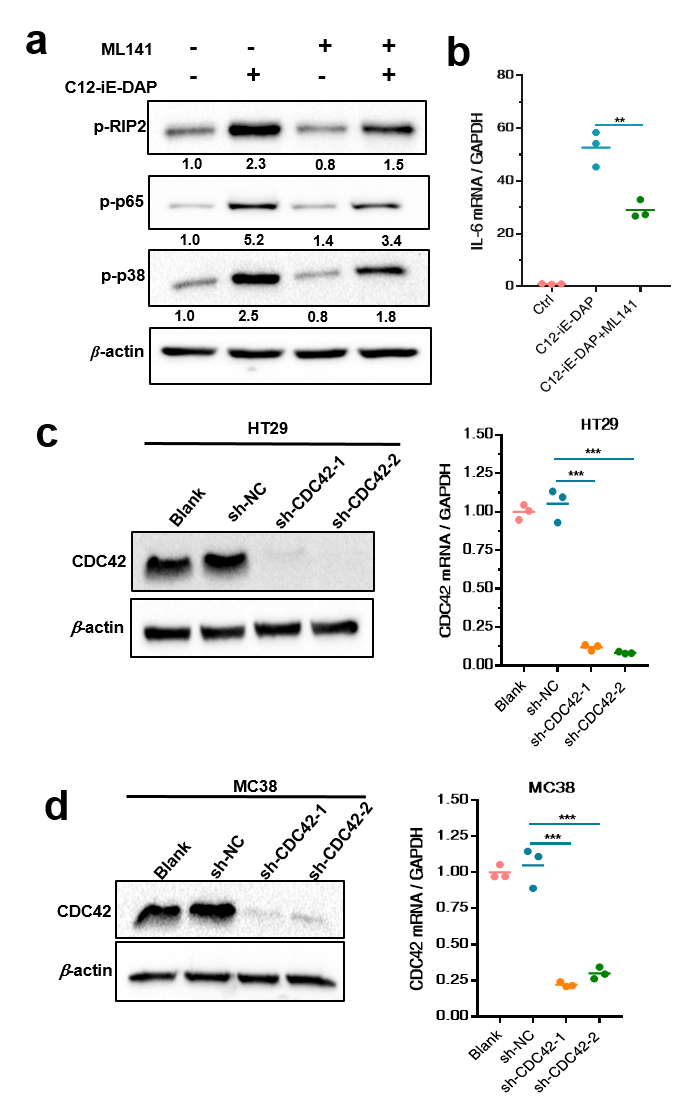


Supplementary Figure 7

*
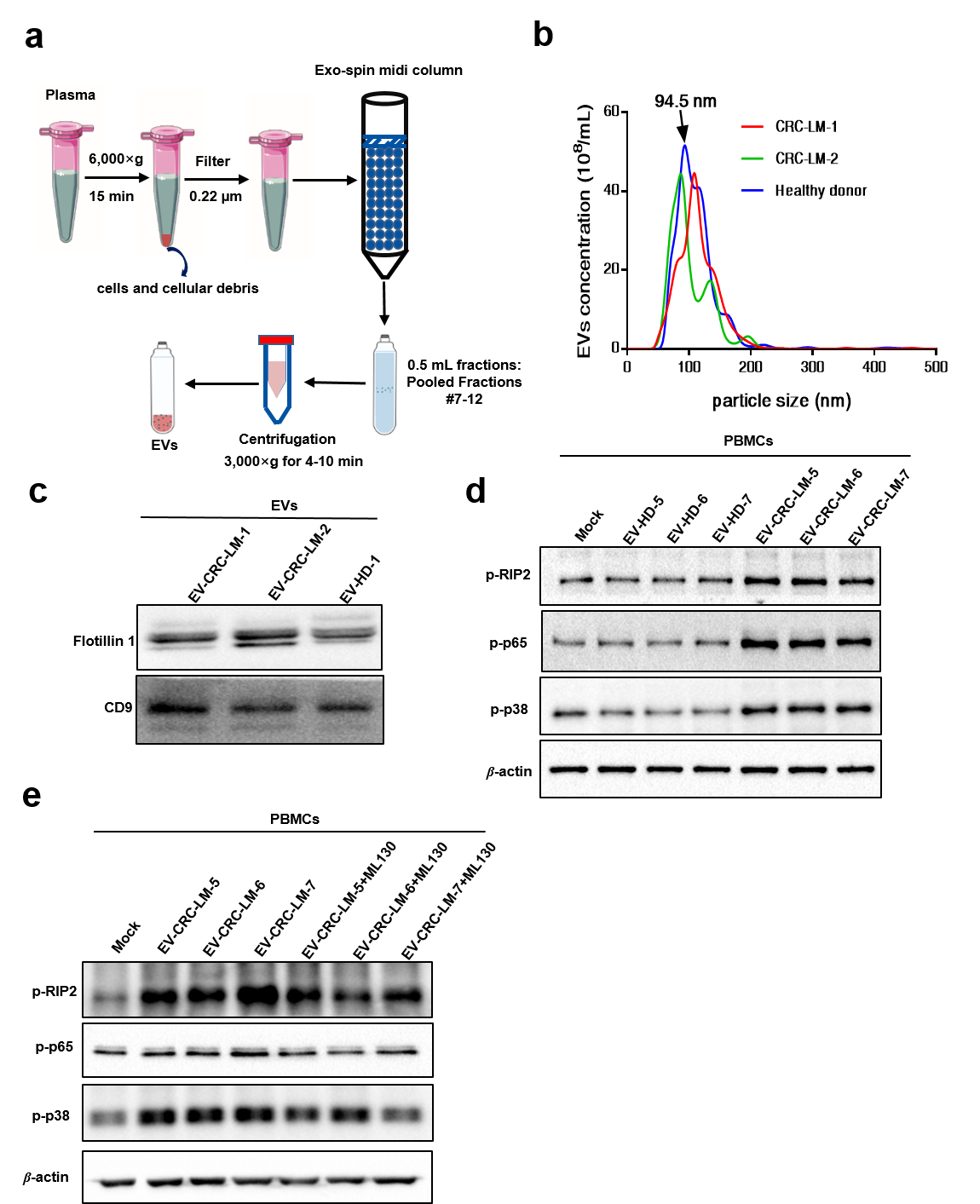
*

**Supplemental Table**

**Supplementary Table 1 Primer sequences used in qPCR**

| **Gene** | **Primer Sequences** |
| --- | --- |
| Human IL-6 | F: 5’-ACTCACCTCTTCAGAACGAATTG-3’  R: 5’-CCATCTTTGGAAGGTTCAGGTTG-3’ |
| Human TNF-α | F: 5’-CCTCTCTCTAATCAGCCCTCTG-3’  R: 5’-GAGGACCTGGGAGTAGATGAG-3’ |
| Human CDC42 | F: 5’-CCATCGGAATATGTACCGACTG-3’  R: 5’-CTCAGCGGTCGTAATCTGTCA-3’ |
| Human CCL1 | F: 5’-CTCATTTGCGGAGCAAGAGAT-3’  R: 5’-GCCTCTGAACCCATCCAACTG-3’ |
| Human CCL2 | F: 5’-CAGCCAGATGCAATCAATGCC-3’  R:5’-TGGAATCCTGAACCCACTTCT-3’ |
| Human GAPDH | F: 5’-CGGAGTCAACGGATTTGGTCGTAT-3’  R: 5’-AGCCTTCTCCATGGTGGTGAAGAC-3’ |
| Human COPE | F: 5’-CAGAGGAAGTTCGGTGTGGTC-3’  R: 5’-GGGCGAGGTAGTCAGCAAAC-3’ |
| Human C1orf43 | F: 5’-ACGCCTTTCAAGGGTGTACG-3’  R: 5’-CAAAGACCCCTGTCCCATAGC-3’ |
| Human ENSA | F: 5’-AGGCCAAATACCCAAGCCTA-3’  R: 5’-GGCCATGTTGTAGTCTCCTGA-3’ |
| Mouse IL-6 | F: 5’-CTGCAAGAGACTTCCATCCAG-3’  R: 5’-AGTGGTATAGACAGGTCTGTTGG-3’ |
| Mouse TNF-α | F: 5’-CTGAACTTCGGGGTGATCGG-3’  R: 5’-GGCTTGTCACTCGAATTTTGAGA-3’ |
| Mouse CDC42 | F: 5’-CCCATCGGAATATGTACCAACTG-3’  R: 5’-CCAAGAGTGTATGGCTCTCCAC-3’ |
| Mouse GAPDH | F: 5’-TGGCCTTCCGTGTTCCTAC-3’  R: 5’-GAGTTGCTGTTGAAGTCGCA-3’ |

**Supplementary Table 2. List of antibodies used in western blotting (WB) and immunofluorescence (IF)**

| **Antibody** | **Source and identifier** | **Dilution** | **Appication** |
| --- | --- | --- | --- |
| Phospho-NF-κB p65(Ser536) | CST (#4511) | 1:1,000 | WB |
| Phospho-p38 MAPK (Thr180/Tyr182) | CST (#4511) | 1:1,000 | WB |
| Phospho-RIP2 (Ser176) | CST (#14397S) | 1:1,000 | WB |
| RIPK2 Antibody (Phospho-Ser176) | Aviva Systems Biology(OAAJ01679) | 1:800 | WB |
| Anti-beta Actin antibody (HRP) | Abcam (ab 49900) | 1:5,000 | WB |
| Anti-CD9 antibody | Abcam (ab 92726) | 1:2,000 | WB |
| Anti-Flotillin 1 antibody | Abcam (ab 133497) | 1:2,000 | WB |
| Anti-CD63 antibody | Abcam (ab 134045) | 1:500 | WB |
| Anti-ALIX antibody | Abcam (ab 186429) | 1:1,000 | WB |
| Anti-Calnexin antibody | Abcam (ab 133615) | 1:1,000 | WB |
| Anti-TSG101antibody | Abcam (ab 125011) | 1:1,000 | WB |
| Anti-CDC42 antibody | Abcam (ab 187643) | 1:1,000 | WB |
| Anti-NOD1 antibody | Abcam (ab 189409) | 1:1,000 | WB |
| Anti-beta Actin antibody | Abcam (ab 6276) | 1:2,000 | WB |
| Goat **A**nti-Mouse IgG (H+L) | ZSGB-BIO (ZB-2305) | 1:5,000 | WB |
| Goat Anti-Rabbit IgG (H+L) | ZSGB-BIO (ZB-2301) | 1:5,000 | WB |
| Alexa Fluor ^TM^ 488 donkey anti-rabbit IgG (H+L) | Invitrogen (A21206) | 1:1,000 | IF |
| Goat Anti-Mouse IgG (H+L) Alexa Fluor TM Plus 647 antibodies | Invitrogen (A32728) | 1:1,000 | IF |
| Anti-NOD1 antibody | Abcam (ab 189409) | 1:50 | IF |
| F4/80 Monoclonal Antibody (BM8) | eBioscience (#14-4801-85) | 1:50 | IF |

**Supplementary Table 3 shRNA target sequence**

| Gene and shRNA target sequence |
| --- |
| Human CDC42 (gene ID:998)  Human-shCDC42-1: 5’-CCGGCCCTCTACTATTGAGAAACTTCTCGAGAAG  TTTCTCAATAGTAGAGGTTTTTG-3’  Human-shCDC42-2: 5’-CCGGCGGAATATGTACCGACTGTTTCTCGAGAAA  CAGTCGGTACATATTCCGTTTTTG-3’  Mouse CDC42 (gene ID:12540)  Mouse-shCDC42-1: 5’-CCGGCGGAATATGTACCAACTGTTTCTCGAGAAA  CAGTTGGTACATATTCCGTTTTTG-3’  Mouse-shCDC42-2: 5’-CCGGAGCCATACACTCTTGGACTTTCTCGAGAAA  GTCCAAGAGTGTATGGCTTTTTTG-3’ |

**Supplementary Table 4 The known small Rho GTPases ^2^**

| Symbol | Protein name |
| --- | --- |
| RHOA | Ras homolog family member A |
| RHOB | Ras homolog family member B |
| RHOC | Ras homolog family member C |
| RHOD | Ras homolog family member D |
| RHOF | Ras homolog family member F, filopodia associated |
| RHOG | Ras homolog family member G |
| RHOH | Ras homolog family member H |
| RHOJ | Ras homolog family member J |
| RHOQ | Ras homolog family member Q |
| RHOU | Ras homolog family member U |
| RHOBTB1 | Rho related BTB domain containing 1 |
| RHOBTB2 | Rho related BTB domain containing 2 |
| RND1 | Rho family GTPase 1 |
| RND2 | Rho family GTPase 2 |
| RND3 | Rho family GTPase 3 |
| CDC42 | Cell division cycle 42 |
| RAC1 | Rac family small GTPase 1 |
| RAC2 | Rac family small GTPase 2 |
| RAC3 | Rac family small GTPase 3 |
| CHP1 | Calcineurin like EF-hand protein 1 |

Soure Data of Western Blotting


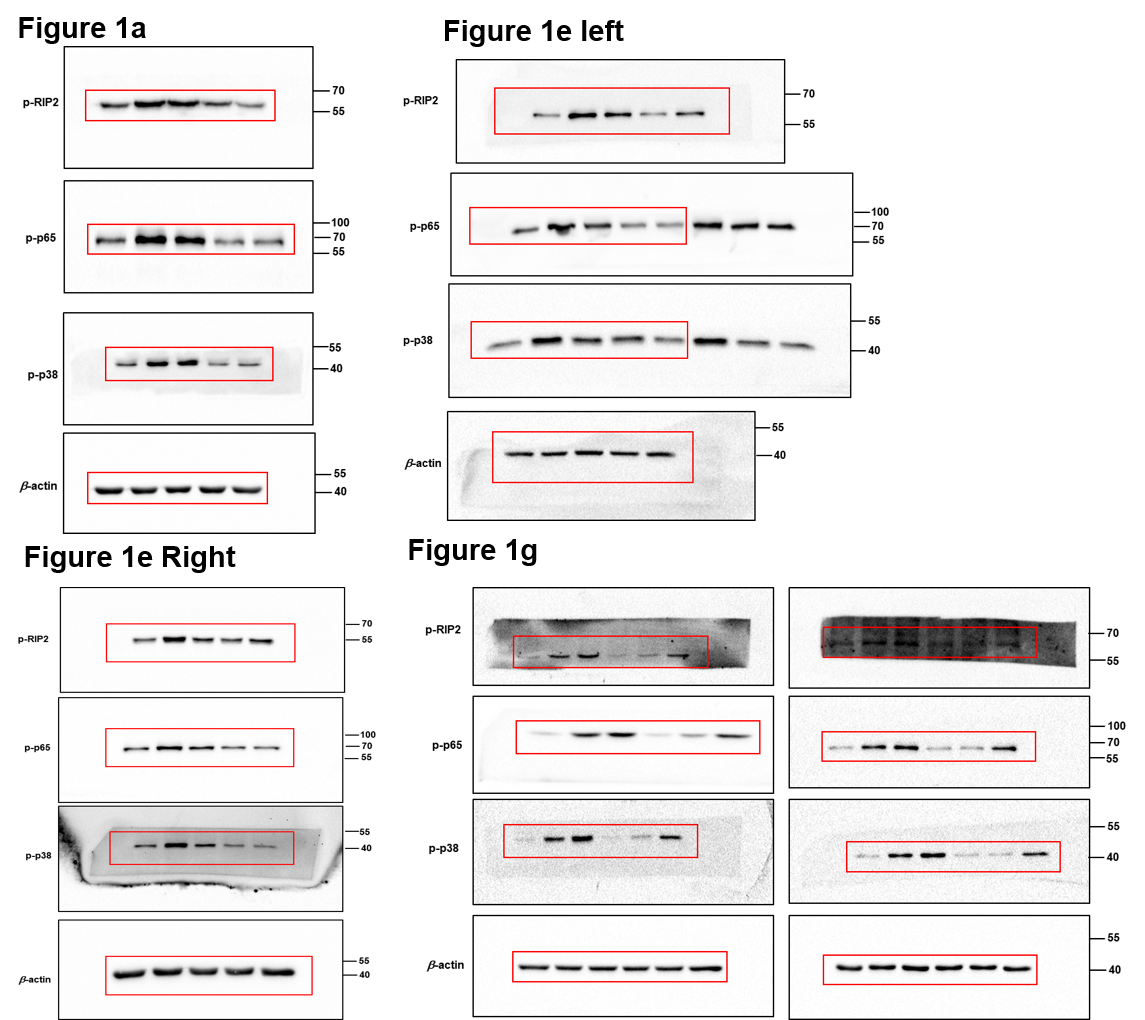


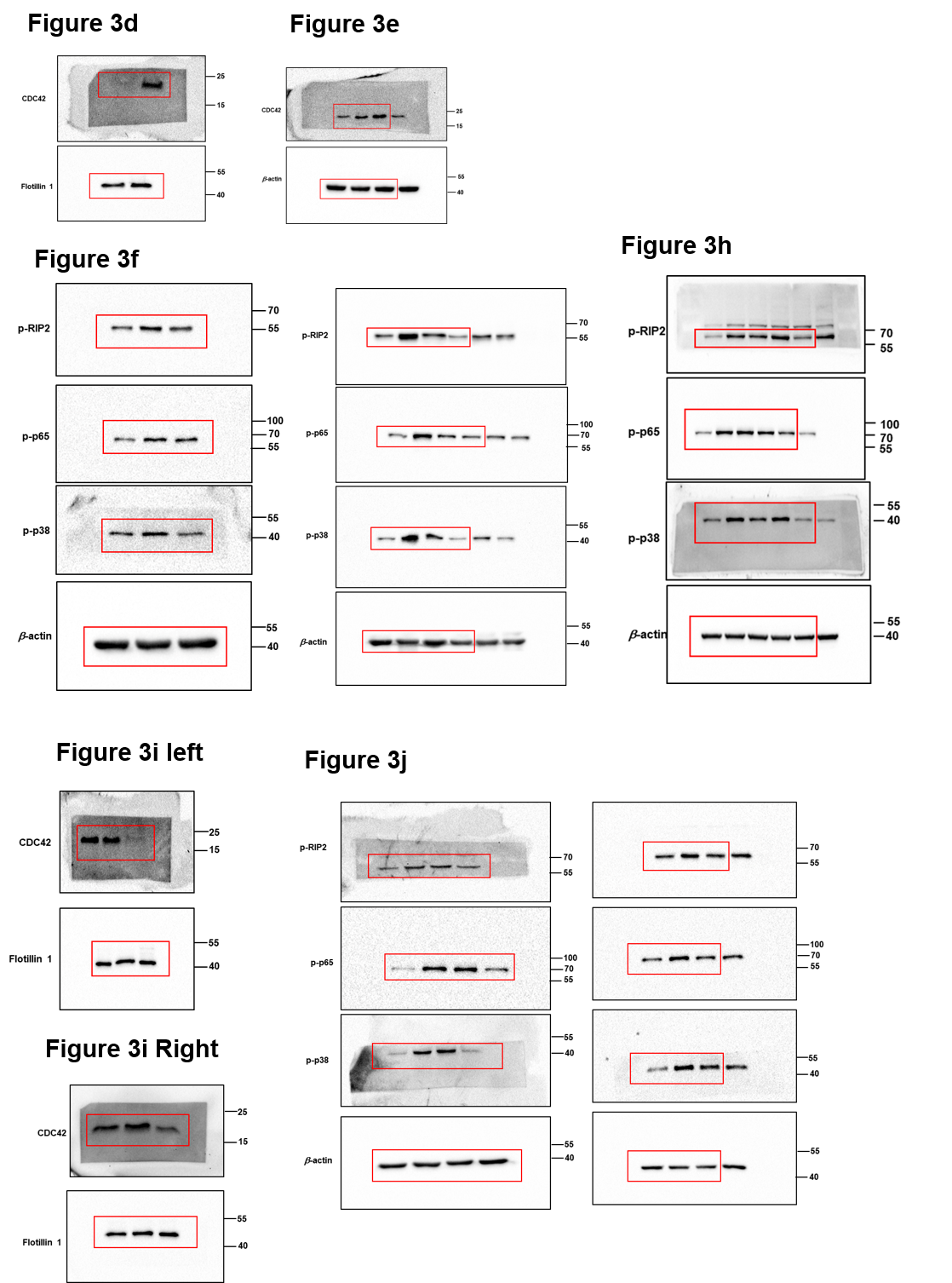


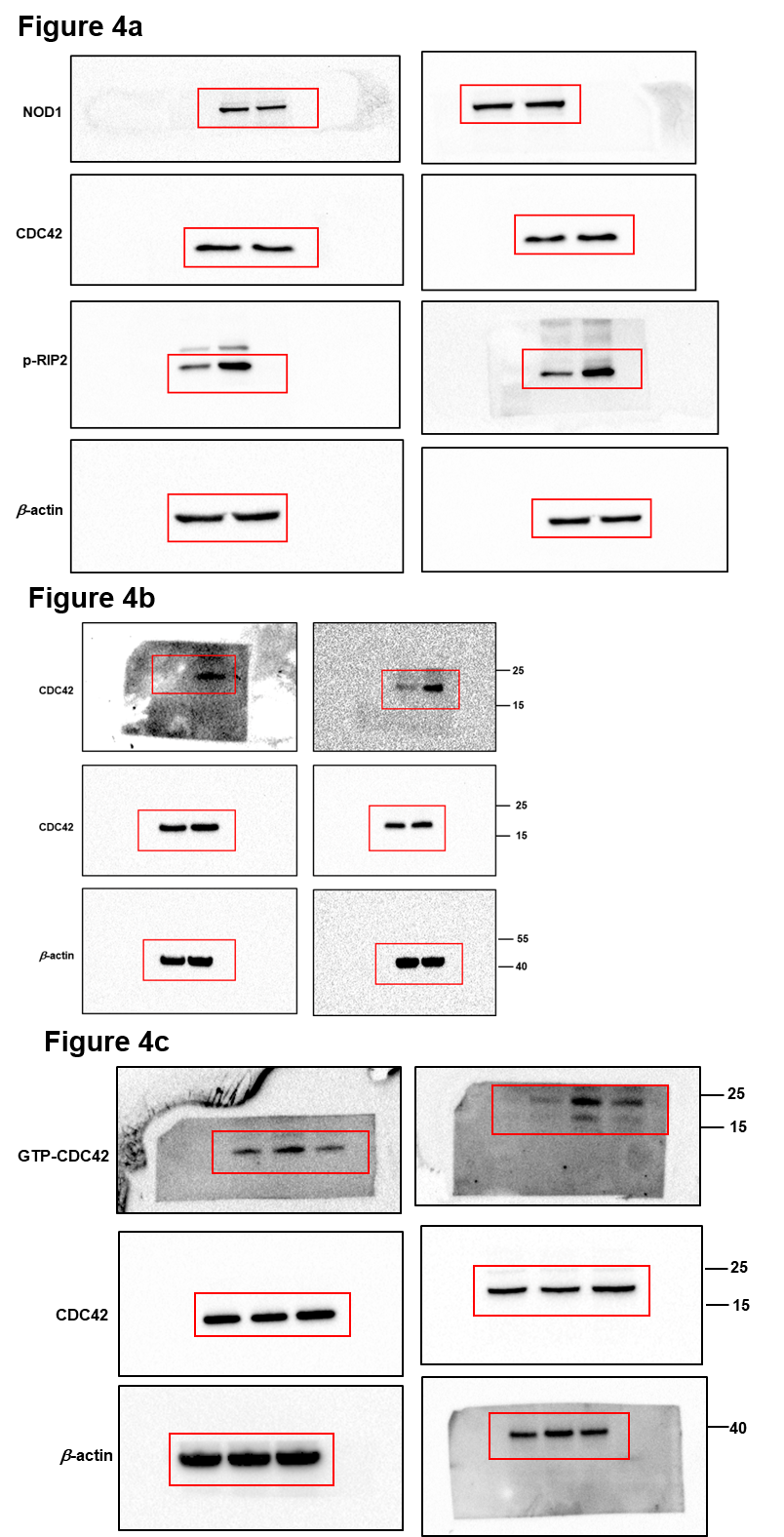


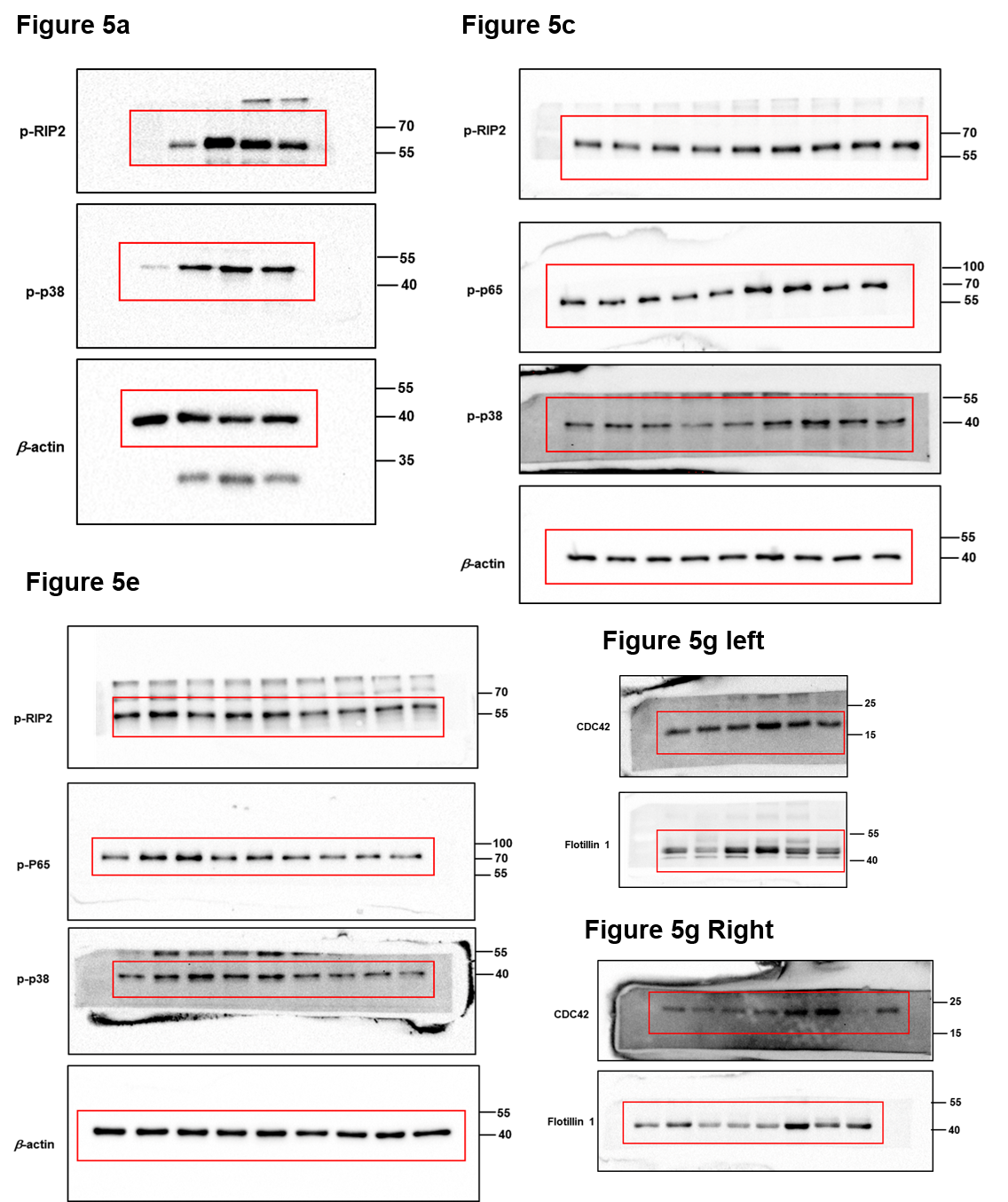


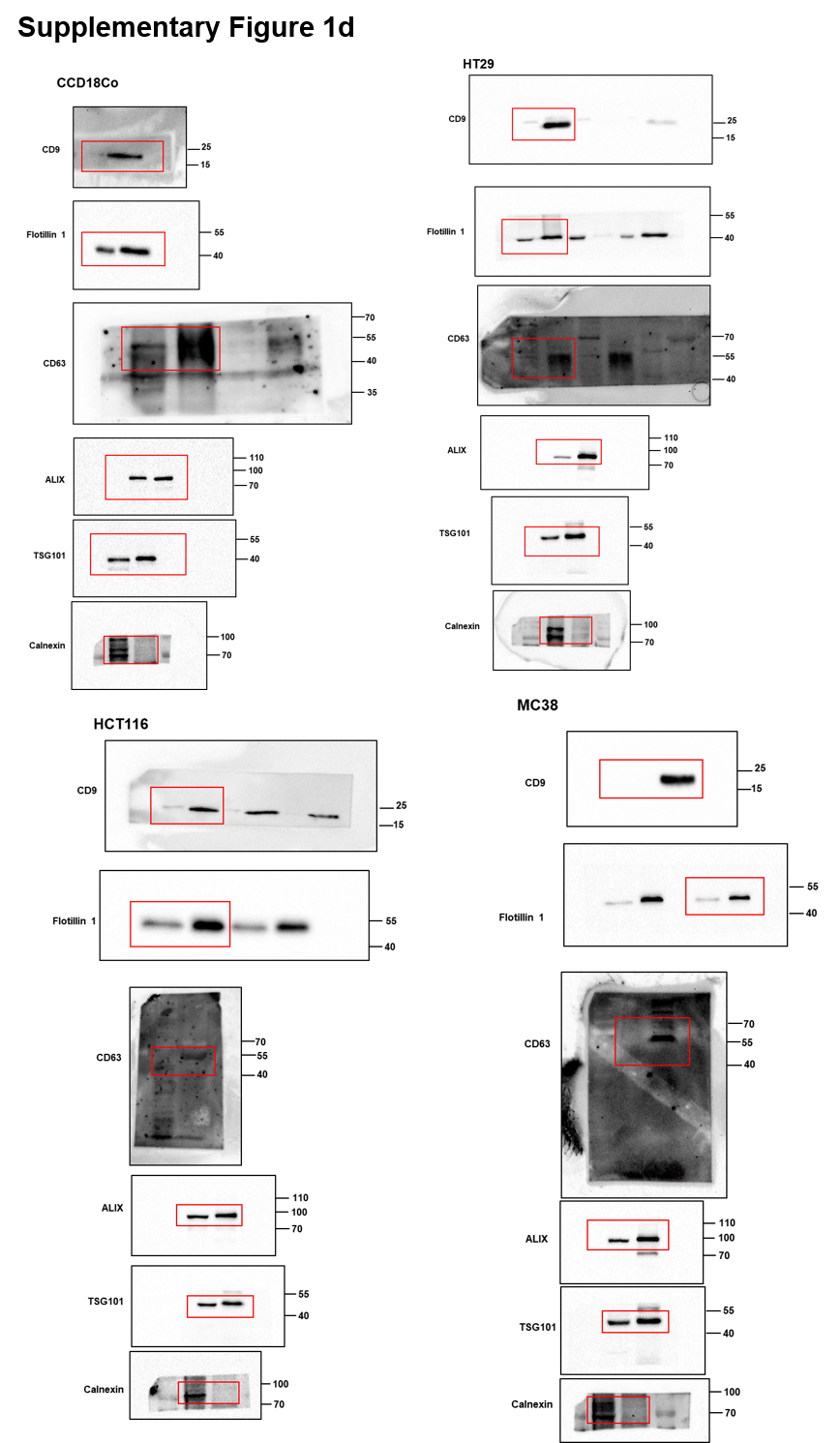


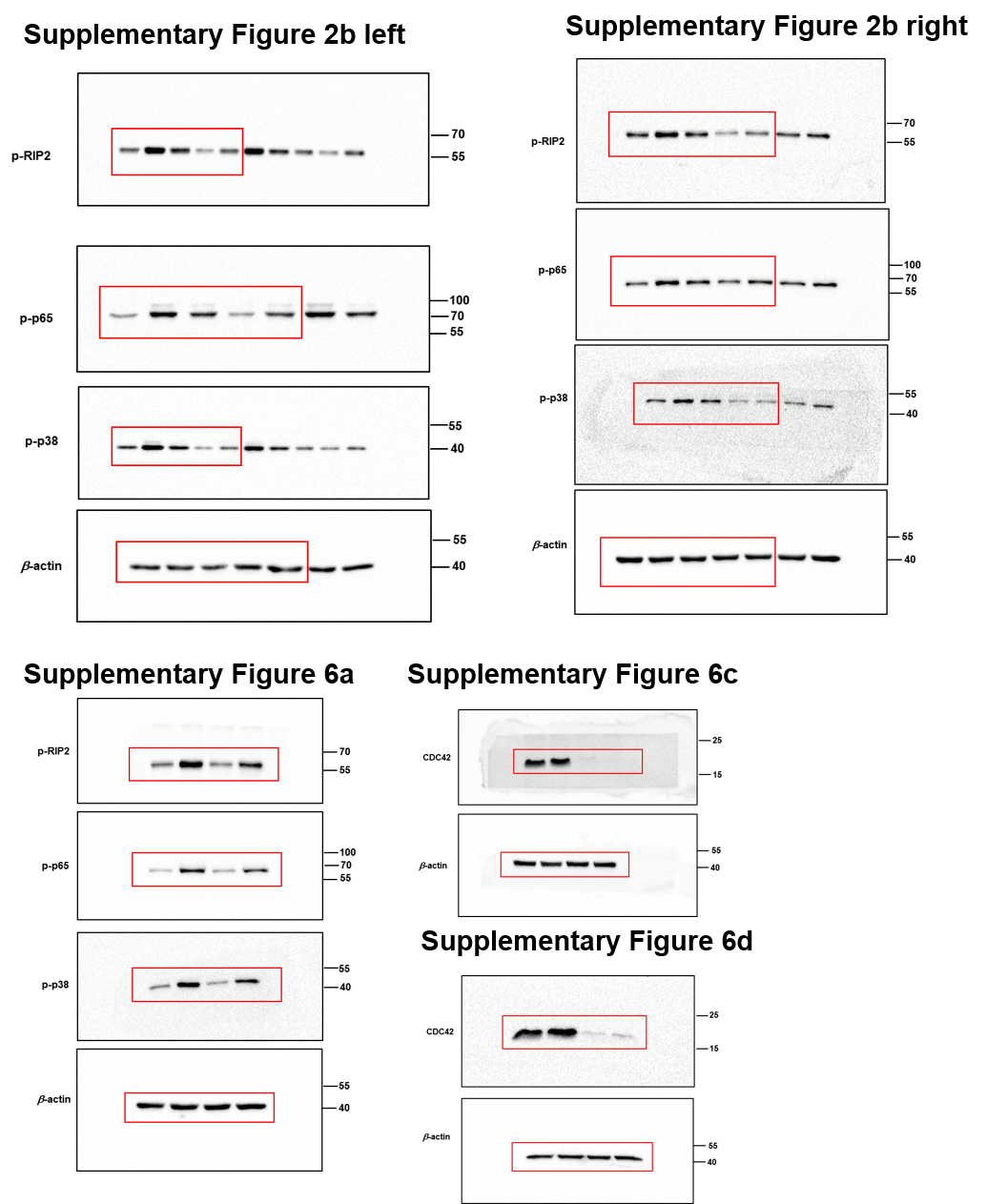


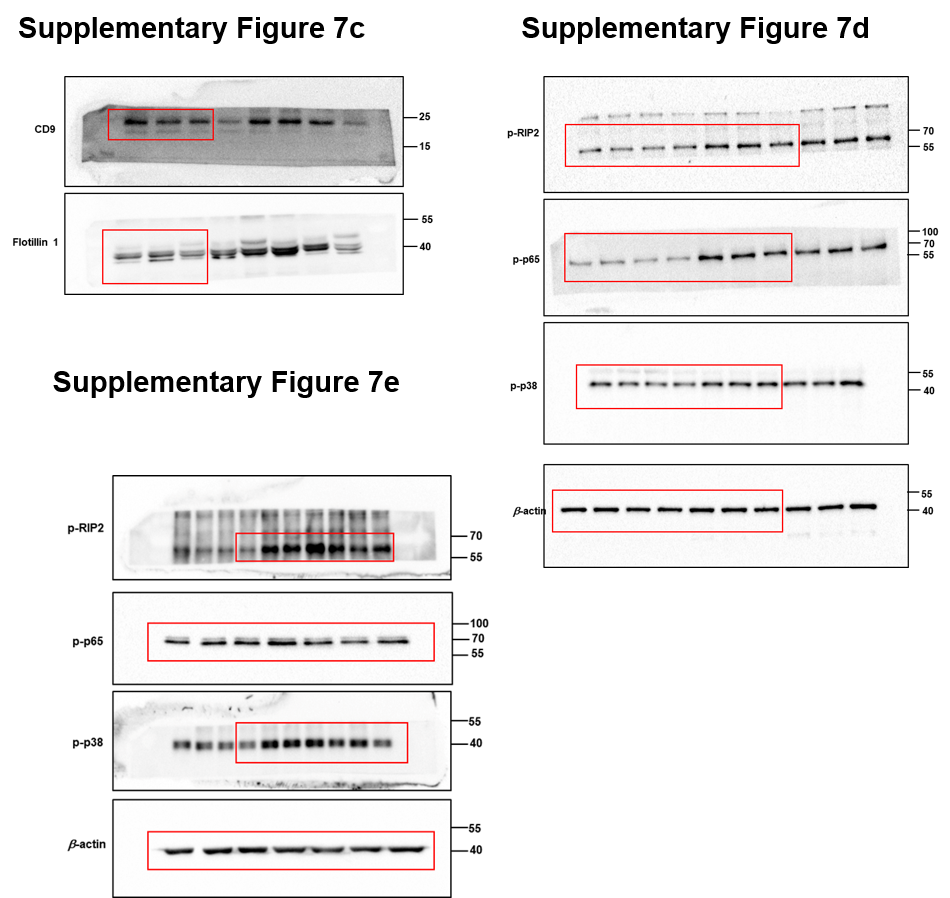

Supplement: Supplementary file 1 — Supporting Information [file JEV2-11-e12264-s001.docx]
